# Effect of group education and person-centered support in primary health care on mental health and quality of life in women aged 45–60 years with symptoms commonly associated with stress: a randomized controlled trial

**DOI:** 10.1186/s12905-023-02221-6

**Published:** 2023-03-24

**Authors:** Lena Rindner, Lena Nordeman, Gunilla Strömme, Dominique Hange, Ronny Gunnarsson, Gun Rembeck

**Affiliations:** 1 Region Västra Götaland, Primary Care, Närhälsan, Södra Torget Health Care Center, Kvarngatan 4, 503 38 Borås, Sweden; 2 Research, Education, Development, Education and Innovation, Primary Health Care, Region Västra Götaland, Sweden; 3 General Practice/Family Medicine, School of Public Health and Community Medicine, Institute of Medicine, Sahlgrenska Academy, University of Gothenburg, Gothenburg, Sweden; 4 Sahlgrenska Academy, Institute of Neuroscience and Physiology Department of Health and Rehabilitation, Unit of Physiotherapy, University of Gothenburg, Gothenburg, Sweden; 5 Region Västra Götaland, Primary Care, Närhälsan, Tidan Health Care Center, Skövde, Sweden; 6 Primary Health Care Clinic for Homeless People, Region Västra Götaland, Sweden

**Keywords:** Menopause, Women’s health, Primary health care, Person-centered, Mental health, Menopause Rating Scale, Incontinence, Education

## Abstract

**Background:**

Mental illness and somatic symptoms are common causes of long-term sick leave for women during menopause, which usually occurs between the ages of 45 and 55. Many women experience a lack of knowledge about menopause and its associated symptoms. This study evaluates the effect of group education and person-centered individual support in primary health care (PHC) on mental health and quality of life for women in menopause with symptoms that are usually associated with stress.

**Methods:**

The randomized controlled clinical trial (RCT) with a two-factor design was conducted in PHC in southwestern Sweden, from 2018 to 2019. A total of 370 women aged 45–60 were allocated in four groups: 1, group education (GE) 2, GE and person-centered individual support (PCS) 3, PCS and 4, control group. GE comprised four weekly sessions and PCS included five sessions with topics related to menopause. The effect of the interventions were followed up at 6 and 12 months. Linear and ordinal regression were used to analyse the effect of the intervention, either group education or person-centred individual support.

**Results:**

The main findings: Improved quality of life and physical, psychological, and urogenital symptoms. GE and PCS resulted in improvement of the quality of life at six months. At the 12-month follow-up these results were significantly strengthened for PCS and improved health-related quality of life, and reduced mental, urogenital, and stress-related symptoms with an effect lasting at least 12 months. These results suggest that this intervention could be an effective intervention in PHC for improving women’s health in menopause.

**Conclusions:**

PCS can be an effective intervention in PHC for improving women’s health in menopause and possibly also prevent the development of exhaustion syndrome.

*Trial registration*: Universal trial number is U1111-1219-6542 and the registration number in ClinicalTrials.gov is NCT03663075, date of registration 10/09/2018.

**Supplementary Information:**

The online version contains supplementary material available at 10.1186/s12905-023-02221-6.

## Introduction

Middle-aged women often enter new life challenges with various health impacts, and it often coincides with menopause transition [1, 2]. From 45 to 60, women's physical and mental health show a marked decrease [3, 4] causing long-term sickness and ill health to a greater extent than men [5]. Moreover, common mental disorders (CMD) such as depression, anxiety and other stress-related illnesses are common causes for attending primary health care (PHC) and need for long-term sick-leave [4, 5].

### Menopausal symptoms

Menopausal symptoms are often linked with symptoms from the vasomotor system, the skeleton, joints, muscles and urogenital tract [1, 3]. Symptoms such as mental exhaustion, sleep disorders and the metabolic syndrome, independent risk factors of developing cardiovascular disease, are increasing during the age 45–60 [1, 6]. Falling estrogen levels and menopause are not necessarily correlated with women's physical and mental symptoms and changes in the 45–60 age group [3]. During this phase in life, which often coincides with menopause, women undergo a hormonal conversion with reduced levels of oestrogen as well as bio-psychosocial changes [1, 3, 7]. Psychosocial factors have a major impact on women's quality of life during menopause [8]. Therefore, it is important to have a person-centered, holistic and a biopsychosocial approach to women's symptoms, health, and life situation and how it affects quality of life.

### Mental health in middle age

CMD is the dominating mental health problem for women and has increased in Sweden as well as in other Organisation for Economic Co-operation and Development (OECD) countries [5, 9] leading to long term sick leave [5]. Stress-related symptoms appear after prolonged high stress levels without adequate recovery [10]. There are currently few studies with strong evidence for treatment of symptoms commonly associated with stress [11]. CMD for women tend to be more common during middle age, which often coincides with the menopause transition (MT) [1]. Furthermore, women often lack information about normal physical, mental and urogenital changes during middle age [7, 12].

### Women’s transition in middle age

During middle age, changes of varying physiological and psychosocial degrees occur. These changes often coincide with changes in life such as personal and social relationships such as caring for ageng parents and teenage children. But also, by major life events such as the death of parents, children leaving home or becoming grandparents [1, 3]. How this period in life experienced is influenced by factors such as lifestyle, attitude and culture [3].

Menopause occurs in average around 51 years of age with a significant global variation [2, 13]. Peri-menopause is a variable period extending 5 to 10 years before and after the final menstruation [13]. Hot flushes, depressed mood and other perimenopausal symptoms can begin well before menstrual irregularities and can continue well beyond the final menstruation [1]. They may affect as many as four out of five women with a varying degree of severity and disruption in their lives [1, 3].

### Interventions

Group education can provide information and offer an opportunity to exchange experiences [14]. Interventions with group education related to middle-aged women’s health showed improvements in knowledge and attitudes towards menopause and improved quality of life [15, 16]. A review showed many publications stating a positive effect, such as improved self-management strategies, knowledge, and peer support, from group education [15].

Person-centered individual support is another option that can raise awareness of available personal choices and their consequences, create awareness into behaviours and provide insight into one´s overall life situation to identify possible explanations for poor health [17]. Health-related quality of life improved faster in patients receiving person-centered support than for those who received treatment-as-usual.

### The remaining dilemma

Today there are few educational opportunities, support and care for middle-aged women going through the normal process of reproductive ageng [12]. However, it is unclear what support measures middle-aged women need and which interventions are effective [7, 12]. There is sparce research on group education and on person-centered individual support related to middle-aged women [12]. Thus, this study aims to evaluate the effect of group education as well as person-centered individual support in a primary health care context on mental health issues and quality of life in women aged 45–60 with symptoms commonly associated with stress.

## Methods

### Study design

The present study is a randomized controlled clinical trial (RCT) with four parallel arms in a two-factor design. The study was conducted in primary health care in Region Västra Götaland in the south-western part of Sweden. Women aged 45–60 with symptoms often associated with stress were recruited from November 2018 until May 2019 by advertisement in local papers.

The study was approved by the Regional Ethical Review Board in Gothenburg Sweden (16/11/2017, registration number 765-17, 2017-11-16). Written informed consent was obtained from all participants and confidentiality was ensured. Trial registration: Universal trial number is U1111-1219-6542 and the registration number in ClinicalTrials.gov is NCT03663075, date of first registration 10/09/2018.

### Participants

Three hundred and seventy women participated in the study. Inclusion criteria were (a) has some form of mental and / or physical health problems such as depression, anxiety, gastrointestinal symptoms, muscular symptoms and/or cardiovascular symptoms, (b) sick leave for a maximum of 30 days during the past two months, (c) understands and can speak Swedish and fill in forms, (d) no severe illnesses such as psychosis, severe depression, or dementia, (e) is not receiving palliative care, (f) has no known current alcohol or substance abuse. Potential participants applied to take part in the study after advertisements in the local press. A research nurse telephoned women expressing an interest in participation and provided further information about the study with an opportunity to ask questions. All women who met the inclusion criteria and accepted participation in the study were included. The exclusion criterium applied after inclusion was new onset of severe psychological stress or illness.

Written informed consent to sign and self-administrated questionnaires regarding health and health-related quality of life were distributed either electronically using the software Esmaker or by ordinary mail.

### Randomization and masking

Research Randomizer was used to generate a random serie based on block-randomization in blocks of four. The random serie was transferred to sequentially numbered closed sealed opaque envelopes, each containing group assignment. Each woman was randomized after she answered all questionnaires at the first assessment. Participants and investigators were not blinded to group allocation due to the nature of the intervention. A total number of 370 women were randomized and allocated into four groups: group 1; Group education (GE), group 2: GE and person-centered individual support (PCS), group 3: PCS and group 4: Control group not receiving any intervention. All participants were informed that they could also seek health care advice wherever they wished outside the scope of the study.

### Intervention

#### The intervention group education (GE)

District nurse and midwife participated as group leaders. Each group included 12–16 women and comprised 1½ hour session per week for four weeks with topics related to physical and mental changes in the body as well as conversations aimed to clarify consequences of behavior, choices in life and to stimulate desired behavioral changes.

The GE was educational, structured and aimed to be person-centered meaning that discussions following education were tailored to questions raised by the group. The topics were biological and physiological processes as well as psychological, emotional, and social aspects of menopause transition. Menopause transition as a natural part of life was emphasized and specific treatment options, pharmacological as well as non-pharmacological, according to the guidelines were clarified. Focus was on factors that prevent disease and promote physical, emotional and social well-being. Furthermore, to provide insight into obstacles and resources, coping strategies and behavioral changes to promote health strategies.

The first session contained education with the definition and myths of menopause transition, physiology, menstrual cycle, hormonal conversion, local oestrogen deficiency symptoms, osteoporosis and information on treatment options, pharmacological and non-pharmacological and self-care advice. The second session contained education and information on cardiovascular health, its risk and health factors, physiology, mental health, relationships, sexuality, and desire as well as information on treatment options, pharmacological and non-pharmacological and self-care advice. The third session contained in-depth insights into mental health, stress-related ill health, physiology, social relationships in family and work, sexuality and desire, insight into obstacles and resources, coping strategies and behavioral changes to promote health. The fourth session contained in-depth insights into relationships, mental health, stress-related illness, health factors as well as a summary of "How do we want to live to feel well?" Insight into obstacles and resources, coping strategies and behavioral changes to promote health.

#### The intervention person-centered individual support (PCS)

Five structured individual person-centered support sessions were carried out by a district nurse or midwife. The topics discussed were the same as in GE but were adapted to the woman’s individual situation and based on the woman´s narratives, needs, resources and beliefs.

The sessions contained a dialogue on symptoms of stress-related ill health, what is happening in the body (physiology), relationships, sexuality, coping strategies, managing demands, requirement, feelings of guilt, exercise, diet, physiology on ageing women, hormonal conversion, menstrual cycle, hot flashes and sweating, local oestrogen deficiency, cardiovascular health, smoking, use of alcohol, sleep and stress. Pharmacological and non-pharmacological treatment options as well as self-care advice were discussed.

PCS was personally tailored focusing on important stressors for that particular woman. PCS specifically focused on necessary changes and “how should I do it” and “how should I think about it” to achieve a better quality of life. It also contained information and discussions about biological and physiological processes as well as psychological, emotional and social aspects of the natural menopause transition. The discussion focused on factors that prevent disease by promoting physical, emotional and social well-being.

First session: Person-centered assessment conversation based on the woman's needs, based on the women´s narratives, needs, resources, beliefs, and goals. A mapping of stressors was performed using the outcome of rating scales (MRS, s-ED, HADS, AUDIT) and mood on a visual analog scale (VAS 1–100 mm) and these were jointly reviewed and discussed. VAS was used as a tool in PCS and is therefore not evaluated as part of the study. If there was a need for pharmacologic treatment or sick leave a physician from PHC was contacted, and treatment was followed up.

The second to fourth sessions were also person-centered. Each session opened with a recap of the previous session. The fifth session contained a summary and discussion of previous sessions and the patient filled in the same rating scales as in the first session, and tools for coping strategies were discussed.

### Data collection

All participants filled a first assessment demographic questionnaire that included age, educational level, family status and work status. Further, visits to PHC and cause (mental and/or physical), menopause status (bleeding pattern and use of hormonal contraception), Menopause Hormone Therapy (MHT), alcohol habits using Alcohol Use Disorders Identification Test (AUDIT) and presence of hypertension were inquired about and participants were asked to fill the following self-administrated validated questionnaires at base-line and at 6 months and 12 months after the intervention.

The women also filled in the following self-administrated validated questionnaires:

- The Short-Form Health Survey (SF-36) version 1 for physical and mental health where a higher value indicates better health [18].
- The Hospital Anxiety and Depression Scale (HADS) to examine anxiety and depressive symptoms [19]. A lower value indicates less depression and anxiety [20].
- The self-rated exhaustion disorders (s-ED) to estimate the risk of developing clinical exhaustion syndrome with reduced work capacity and increased risk of sick leave [10]. Higher scores indicate a higher level of risk of developing a clinical exhaustion syndrome.
- The Perceived Stress Scale (PSS-14) registered general perceived stress, and higher scores indicate a higher level of symptoms [21].
- The Montgomery-Asberg Depression Rating Scale (MADRS-S) was used for depression where a higher score indicates more severe symptoms [22].
- The Menopause Rating Scale (MRS) was used for evaluation of the prevalence and severity of aging signs and their impact on the health-related quality of life as developed by Heinemann and validated in Sweden [23]. The MRS questionnaire consisting of eleven items, divided into three subscales reflecting; somatic symptoms—hot flushes, chest discomfort (irregular heart rhythm or feeling extra heart beats), sleeping problems and muscle and joint problems; psychological symptoms—depressive mood, irritability, anxiety and physical and mental exhaustion; and urogenital symptoms—sexual problems, bladder problems and vaginal dryness. Higher score indicates more severe symptoms [23].

Work status was registered as currently working/studying, sick leave full-time, sick leave part-time, disability pension (full-time), disability pension (part-time), unemployed full-time or unemployed part-time. Cause and visit to PHC was registered for the last two months.

For registration of menopausal status the criteria of the Stages of Reproductive Aging Workshop was used, comprising criteria for; premenopausal women having regular menses, and perimenopausal irregularities > 7 days from their normal cycle and postmenopausal no menses in the last 12 months [13]. Treatment with MHT was registered as local or systemic administration or not using MHT. Having a hormonal spiral (IUD) or other hormonal contraceptive affecting bleeding patterns was also registered.

Presence of known high blood pressure was registered: never had high blood pressure, or had high blood pressure only during previous pregnancy, or think they have previously had hypertension unrelated to pregnancy, or currently have high blood pressure but is not medicated for this, or have high blood pressure and is currently taking medication for this.

The effect of the interventions were followed up at 6 and 12 months after the first assessment where AUDIT, SF36, HADS, s-ED, PSS-14, MADRS-S and MRS questionnaires were repeated.

The primary outcome was the effect of GE on quality of life measured by SF-36 at the 6-month follow-up. Secondary outcomes were if GE also had any effect on other aspects of health-related quality of life measured by HADS, s-ED, PSS-14, MADRS-S and MRS at 6 months. Another secondary outcome was if GE or PCS had an effect on any of these aspects of health-related quality of life at the 12-month follow-up.

### Statistical analysis

The first assessment demographic data and self-administered questionnaires were presented with mean and standard deviation (SD), median and interquartile range (IQR), number and percent (%) dependent on data level. Sum scores for all self-administered questionnaires: SF-36, HADS, s-ED, PSS-14, MADRS-S and MRS were calculated according to their respective manuals.

### Change over time

Change between the first assessment and the 6- and 12-month follow-up was calculated. Nominal data, such as changes in work status, visit to PHC and MHT administration, menopause status and AUDIT are presented as number (%) at the first assessment and at follow-up. Other changes in questionnaires are presented as mean change (SD). The change between the first assessment and follow-up were also categorised into three categories, worsening, unchanged or improved (coded as − 1, 0, + 1).

### Inferential statistics

Inferential statistical analysis was a two-factor design repeated for intention to treat (ITT), complete case (CC) and per protocol (PP) analysis. ITT; all participants included with last outcome carried forward for missing data (LOCF). CC; participants who responded to the follow-up survey and PP; participants who received allocated intervention and responded to the follow-up survey. The level of significance was set to 0.05. The IBM SPSS Windows version 25 and 27 was used for statistical analyses.

For the effect of either intervention at 6- and 12-month follow-up linear and ordinal regression were used. The first linear regression was performed with mean change in SF-36, HADS, s-ED, PSS-14, MADRS-S and MRS as the dependent variable. The mean changes were not normally distributed, so they were transformed using Blom’s rank-based method [24]. Independent variables were GE, PCS and the interaction between GE and PCS. The Beta coefficient in the linear regression cannot be interpreted for ranked data but provides information about direction of effect. The assumptions for linear regression were checked for multicollinearity (Additional file 1: Tables S2 and S4) and the linear regression models were tested for heteroskedasticity. Secondly ordinal regressions were performed. Transformed change in outcome variables, coded as worsening − 1, unchanged as 0 and improvement + 1, was used as the dependent variable. Independent variables were GE, PCS and interaction between GE and PCS. All regression analyses were adjusted for age.

Finally, a summary of the results from linear and ordinal regression analyses were presented in a summary table. In the summary table a desired effect of any of the two interventions was denoted with a + sign if it was statistically significant in at least one of ITT, CC and PP. A double + sign indicates a desired effect of intervention seen in two of the three statistical analysis and three + signs indicate a statistically significant effect in all three analysis. In analogue with this 1–3 minus signs indicate a statistically significantly undesired effect of the intervention. The odds ratio is also presented for any statistically significant effect seen in the ordinal regression of the ITT analysis. It should be noted that some questionnaires estimate health while others estimate symptoms. Hence, a desired effect in some outcome variables result in an odds ratio > 1 while in others an odds ratio < 1. To enhance understanding this was transformed in the summary table, so a desired effect always had an odds ratio > 1 while an undesired effect always had an odds ratio < 1.

### Sample size calculation and power

When estimating sample size we assume a level of significance of 0.05, a power of 0.80 and a two-tailed test. We used simple group comparison for two independent groups with Mann–Whitney's test as surrogate test. The sample size estimation recommends including 368 women (for details see Additional file 1). It was our intention to include 370 women.

## Results

**Fig. 1:**
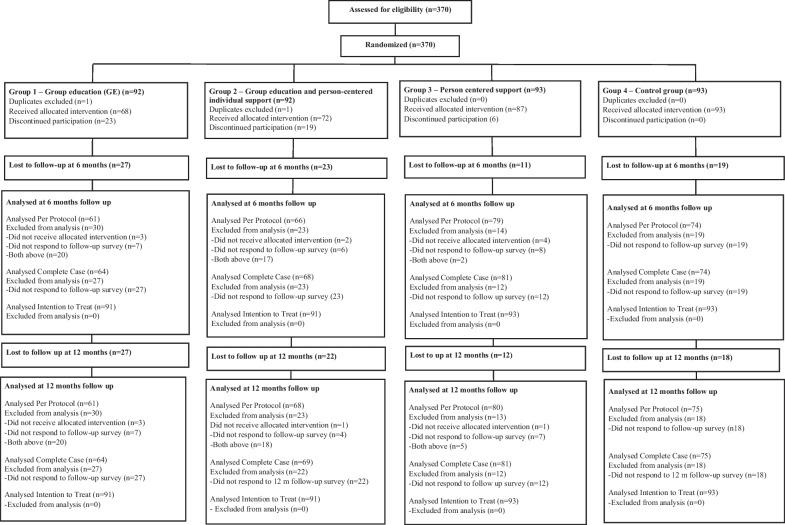
Participant flow

Three hundred and seventy women were included in the study (Fig. 1). Two women were mistakenly included twice and were excluded. Of the 368 remaining, 287 (78%) participated in the follow up at six months and 289 (79%) at 12 months. Most of those lost to follow-up in group 1–3 were participants discontinuing their allocated intervention. Some women lost to follow up at 6 months participated in the 12 months follow-up.

For the total group (n = 368) the average age was 52 (SD 7.0) years (Table 1). Half of the women had tertiary school education 188/368 (51%). Most of the women (94%) were at work, living with a partner (79%) and had children living at home (55%). The majority of women (90%) had non risky alcohol consumption. Eighty-five percent (312/368) of the women were not using MHT (Table 1).

**Table 1:** Patient characteristics at first assessment

	Group 1—GE		Group 2—GE + PCS		Group 3—PCS		Group 4—Control		Total n = 368	
	n = 91		n = 91		n = 93		n = 93			
Age (y)^a^	53 (4.2)	53 (7.0)	53 (3.7)	53 (5.0)	53 (4.2)	53 (7.0)	51 (3.9)	51 (6.0)	52 (4.0)	52 (7.0)
Education (y)^b^										
Primary/Secondary school (< 12 year)	46 (51)		49 (54)		48 (52)		36 (39)		179 (49)	
Tertiary school (> 12 year)	45 (49)		41 (46)		45 (48)		57 (62)		188 (51)	
Work status/employment status^b,c^										
Currently working	77 (85)		79 (88)		75 (81)		83 (89)		347 (94)	
Sick leave full-time/part-time	6 (7)		8 (9)		12 (13)		7 (8)		33 (9)	
Disability pension	7 (8)		3 (3)		4 (4)		2 (2)		16 (4)	
Family status^b^										
Living with a partner	67 (74)		73 (81)		73 (79)		75 (81)		288 (79)	
Widow	0 (0)		0 (0)		2 (4)		1 (2)		3 (2)	
Children at home	47 (52)		44 (48)		47 (51)		63 (68)		201 (55)	
Visit PHCC last 2 month^b^	39 (43)		35 (39)		46 (50)		46 (50)		166 (45)	
Cause of recent visit PHCC^b^										
Physical symptoms/complaints	55 (60)		51 (56)		48 (52)		51 (55)		205 (56)	
Psychological symptoms/complaints	18 (20)		23 (25)		19 (20)		17 (18)		77 (21)	
*Menopause*										
MHT (Menopause Hormone Therapy)^b^										
MHT (treatment system tablet)	6 (7)		8 (9)		6 (7)		6 (7)		26 (7)	
MHT (treatment local)	10 (11)		7 (8)		7 (8)		6 (7)		30 (8)	
MHT not use	75 (82)		76 (83)		80 (86)		81 (87)		312 (85)	
MHT use	16 (18)		15 (17)		13 15)		12 14)		56 (15)	
Hypertension^b^										
Never measured blood pressure	0 (0.0)		4 (4.4)		0 (0.0)		1 (1.1)		5 (1.4)	
Have high blood pressure during pregnancy, but not otherwise	6 (7.0)		7 (8.0)		5 (5.4)		6 (7.0)		24 (7)	
Had high blood pressure, but not now	9 (10)		8 (9)		19 (20)		13 (14)		49 (13)	
Have high blood pressure, but do not take any medication for this	2 (2)		2 (2)		6 (7)		3 (3)		13 (4)	
Have high blood pressure and take medication for this	14 (15)		6 (7)		11 (12)		8 (9)		39 (11)	
Never had high blood pressure			63 (70)		52 (56)		62 (67)		237 (64)	
AUDIT^b,h^										
Alcohol habits^a,d^	3.4 (3.2)	3.0 (2.0)	3.0 (2.3)	3.0 (2.0)	3.2 (2.4)	3.4 (3.0)	2.7 (1.9)	2.0 (3.0)	3.0 (2.5)	3.0 (3.0)
Alcohol risk use^b,d^	8 (9)		12 (13)		11 (12)		7 (8)		38 (10)	
*Health related quality of life*										
SF-36^a.e^										
Physical function (PF)	85 (17)	90 (25)	85 (19)	90 (15)	84 (20)	90 (25)	86 (20)	95 (15)	85 (19)	90 (20)
Role physical (RP)	65 (40)	90 (75)	69 (36)	75 (50)	60 (42)	75 (100)	68 (39)	100 (75)	65 (40)	75 (75)
Bodily pain (BP)	63 (28)	62 (43)	64 (25)	62 (42)	58 (26)	62 (28)	62 (25)	62 (43)	62 (27)	62 (43)
General health (GH)	64 (21)	65 (27)	61 (22)	67 (21)	62 (24)	65 (37)	62 (20)	62 (27)	62 (22)	64 (29)
Vitality (VT)	43 (22)	45 (35)	41 (20)	40 (30)	42 (25)	40 (40)	43 (22)	45 (30)	42 (22)	45 (30)
Social function (SF)	65 (27)	63 (38)	67 (27)	75 (38)	64 (28)	62 (38)	71 (24)	75 (44)	67 (27)	75 (38)
Role emotional RE	62 (40)	67 (67)	58 (39)	67 (67)	60 (44)	67 (100)	65 (41)	100 (67)	61 (41)	67 (67)
Mental health (MH)	63 (20)	64 (32)	61 (19)	64 (28)	61 (22)	64 (36)	66 (18)	68 (26)	63 (20)	64 (32)
PCS	48 (11)	50 (17)	48 (11)	50 (13)	46 (12)	49 (15)	47 (11)	51 (14)	47 (11)	50 (15)
MCS	37 (13)	37 (22)	36 (12)	37 (19)	37 (14)	38 (26)	39 (12)	41 (49)	37 (13)	38 (21)
HADS^a,f^										
Depression	5.0 (3.9)	4.0 (5)	5.7 (3.9)	5.0 (7)	5.2 (4.2)	5.0 (7)	4.5 (3.2)	4.0 (5)	5.1 (3.8)	4.5 (6)
Anxiety	7.3 (4.1)	7.0 (6)	7.4 (4.6)	7.0 (7)	7.1 (4.3)	6.0 (6)	7.0 (4.1)	7.0 (6)	7.2 (4.3)	7.0 (6)
s-ED^a,g^	6.0 (3.9)	7.0 (5)	6.0 (2.3)	7.0 (3)	6.0 (2.7)	7.0 (5)	6.0 (2.5)	7.0 (5)	6.0 (2.6)	7.0 (5)
PSS 14^e,h^	25 (9.2)	26 (14)	26 (8.9)	25 (12)	25 (8.9)	26 (12)	23 (8.1)	24 (10)	25 (8.8)	25 (12)
MADRS-S^b,i^	12 (8.4)	12 (12)	13 (8.4)	12 (10)	12 (7.7)	12 (10)	10 (6.4)	10 (8)	12 (7.8)	12 (10)
MRS^b,j^										
Somatic	6.0 (3.8)	6.0 (6)	5.6 (3.1)	5.0 (5)	5.3 (3)	5.0 (5)	5.4 (3.0)	5.0 (5)	5.6 (3.2)	5.0 (5)
Urogenital	3.7 (2.8)	4.0 (5)	4.1 (2.9)	4.0 (4)	4.0 (2.7)	4.0 (4)	3.4 (2.7)	3.0 (5)	3.8 (2.8)	4.0 (5)
Psychological	5.9 (3.8)	5.5 (6)	6.4 (3.7)	6.0 (6)	5.9 (3.8)	6.0 (7)	5.3 (3.7)	4.0 (5)	5.9 (3.7)	5.0 (6)
Total	16 (8.8)	15 (13)	16 (8.2)	16 (11)	15 (7.5)	16 (10)	14 (7.4)	15 (9)	15 (8.0)	16 (11)

^a^First figure Mean (SD), second figure Median (interquartile range)

^b^n %

^c^Work status, work more than one hour/week

^d^AUDIT—alcohol habits, identify risky use, harmful use or alcohol dependence, score ≥ 6 points indicated risk use

^e^SF-36, measure Health-Related Quality of Life. Physical component score (PCS) and mental component score (MCS) separately along with eight domains of SF-36, 0–100 points. Higher score indicated better Health Related Quality of Life

^f^Hospital Anxiety and depression scale (HADS). Higher score indicates increased in anxiety and depression reporting. Cut of score > 8 points in anxiety and depression

^g^Self-rated Exhaustion Disorder (s-ED) identify risk that or may be about to develop a clinical fatigue syndrome with reduced ability to work and increased risk of sick leave. Higher score indicates decrease Quality of Life. Score ≥ 6 indicated fatigue syndrome

^h^Perceived Stress Scale 14 (PSS 14)—measures of mental stress, the degree to which one experiences one's life as unpredictable, uncontrollable, and overloaded. Higher score indicates increased mental stress, highest score 56 points. There are none related cut of score

^i^Montgomery-Asberg Depression Rating Scale (MADRS-S) scoring. Higher score indicates more severe symptoms. First figure mean (SD) second figure median (interquartile range). International standards; 0–6 p no depression, 7–19 p, mild depression, 20–34 p moderate depression, > 34 p severe depression

^j^Menopause Rating Scale (MRS) measure prevalence and severity of symptoms commonly seen during the menopausal transition. Higher score indicates more severe symptoms and worsening Health Related Quality of Life. First figure mean (SD) second figure median (interquartile range). Degree of severity of symptom in the MRS and its domains indicated; Psychological domain; No, little (0–1), Mild (2–3), Moderate (4–6), Severe (7+), Somatic domain; No, little (0–2), Mild (3–4), Moderate (5–8), Severe (9+), Urogenital domain; No, little (0), Mild (1), Moderate (2–3), Severe (4+), Total score; No, little (0–4), Mild (5–8), Moderate (9–16), Severe (17+). MRS subscale: Somatic symptoms—hot flushes, heart discomfort, sleeping problems and muscle and joint problems, Psychological symptoms—depressive mood, irritability, anxiety and physical and mental exhaustion, Urogenital symptoms—sexual problems, bladder problems and vaginal dryness, Total score—all subscales added

The SF36-PCS and SF36-MCS mean values were 47 (SD 11) and 37 (SD13) points respectively, indicating near normal (for PCS) and lowered (for MCS) age-related quality of life. For s-ED the median were 7 points indicating a noticeable risk for developing a clinical exhaustion syndrome (Table 1). The MADRS-S the first assessment values (median 12) indicate mild depression. The values for MRS total, psychological, and somatic were reported as moderate, almost severe discomfort and MRS urogenital indicated severe discomfort (Table 1).

### Changes in health at 6 months

The changes in health at 6 months indicated a reduction in mental, physical, and urogenital symptoms (SF36, HADS, s-ED, PSS-14, MADRS-S, MRS) in all groups (Additional file 1: Table S1a).

### Changes in health at 12 months

The changes in health (SF36, HADS, s-ED, PSS-14, MADRS-S, MRS) indicated an improvement in mental, physical and urogenital symptoms in group 2, 3, 4. Group 1, indicated a decreased health in the domains physical function (PF in SF-36) bodily pain (BP in SF-36) and physical component score (PCS in SF-36) (Additional file 1: Table S1b).

At the 12-month follow-up half of the women 149/289 (49%) had not had any period within the last 12 months. Twelve percent (36/289) had regular menstruation and 18% (52/289) had irregular bleeding within the last 12 months. Twelve percent (36/289) used hormone contraceptives that may influence the bleeding pattern (Additional file 1: Table S1b).

### Effect of GE and PCS at 6 and 12 months

GE had a statistically positive effect, mainly in the area of symptoms measured by the MRS, at 6 months but this effect was lost at the 12-monthfollow-up (Table 2 and Additional file 1: Tables S2–S5). PCS showed a statistically significantly positive effect at 6 months that was further reinforced at the 12-month follow-up (Table 2 and Additional file 1: Tables S2–S5). GE combined with PCS gave an additionally positive effect in physical functioning (PF) and MADRS-S at 6 months that did not remain at the 12-month follow-up (Table 2 and Additional file 1: Tables S2–S5).

**Table 2:** Effect of group education and person-centered individual support at 6- and 12-month follow-up (n = 368)

	Group education (GE)				Person-centered individual support (PCS)				Interaction between GE and PCS			
	6 months		12 months		6 months		12 months		6 months		12 months	
	Linear^a^ regression	Ordinal^a,b^ regression	Linear^a^ regression	Ordinal^a,b^ regression	Linear^a^ regression	Ordinal^a,b^ regression	Linear^a^ regression	Ordinal^a,b^ regression	Linear^a^ regression	Ordinal^a,b^ regression	Linear^a^ regression	Ordinal^a,b^ regression
SF-36^c^												
Physical function (PF)									+ +	+ + + 2.2 (1.0–5.0)		
Role Physical (RP)												
Bodily Pain (BP)			− −	− −								
General Health (GH)			−	−					+ +			
Vitality (VT)							+	+ 1.9 (1.1–3.3)	+	+ +		
Social function (SF)							+ +					
Role emotional (RE)												
Mental health (MH)	+ +					+ + + 1.9 (1.1–3.3)	+ + +	+ + + 2.2 (1.3–4.1)				
PCS			+					+ 1.0 (1.0–1.1)		+ +		
MCS							+ + +	+ + + 2.3 (1.3–4.9)				
HADS^d,i^												
Depression												
Anxiety												
s-ED^e,i^					+	+ 1.9 (1.0–3.7)						
PSS-14^f,i^							+ + +	+ + + 2.5 (1.4–4.5)				
MADRS-S^g,i^	−	− − − 0.33 (0.20–0.63)				−	+ + +	+ 1.7 (1.6–3.0)		+ + + 3.0 (1.1–6.0)		
MRS^h,i^												
Somatic	+ ++	+ + + 2.0 (1.2–3.3)										
Urogenital	+ + +	+ + + 2.0 (1.2–3.3)			+ + +	+ + + 2.6 (1.5–4.5)	+ + +	+ + + 2.4 (1.4–4.1)				
Psychological	+ +				+ + +	+ + + 2.3 (1.3–4.1)	+ + +	+ + + 3.0 (1.9–5.5)				
Total	+ + +	+ + + 2.2 (1.3–3.7)			+ + +	+ + + 2.5 (1.4–4.5)	+ + +	+ + + 3.3 (1.8–5.5)				− 0.43 (0.20–0.54)

^a^A plus or minus sign show statistically significance for either: Per Protocol (PP), Complete Case (CC), or Intention To Treat (ITT) analysis. Plus indicates a desired effect and a minus sign an undesirable effect of the intervention

^b^Second line shows the Odds ratio (95% CI) as the effect size for the effect of intervention found in the ITT analysis if it was statistically significant. An odds ratio above 1 indicates a desirable effect and an odds ratio below 1 an undesirable effect

^c^Short form health survey (SF-36). Physical component score (PCS) and mental component score (MCS)

^d^Hospital Anxiety and Depression Scale (HADS)

^e^Self-rated Exhaustion Disorder (s-ED) identifies risk to develop exhaustion disorder with reduced workability and increased risk of sick leave

^f^Perceived Stress Scale 14 (PSS-14). Identifies degree of mental stress

^g^Montgomery-Asberg Depression Rating Scale (MADRS-S) scoring estimating degree of depression

^h^Menopause Rating Scale (MRS) measure prevalence and severity of aging signs and Health Related Quality of Life. MRS subscale: Somatic symptoms—hot flushes, heart discomfort, sleeping problems and muscle and joint problems, Psychological symptoms—depressive mood, irritability, anxiety and physical and mental exhaustion, Urogenital symptoms—sexual problems, bladder problems and vaginal dryness, Total score—all subscales added

^i^SF36 measures health and hence an increase (odds ratio > 1) is desirable. In HADS, s-ED, PSS-14, MADRS-S, MRS measures symptoms (absence of health) and an increase is not desirable. To facilitate understanding the OR (95% CI) for questionnaires measuring ssymptoms were transformed as odds ratio > 1 indicate a desired change (reduction in symptoms)

## Discussion

The main finding was that PCS resulted in an improvement on health-related quality of life, in women 45–60 with symptoms commonly related with stress lasting at least 12 months after intervention. Hence, this study suggests that PCS, but not GE, can be a low-cost effective intervention in PHC for improving women’s health and possibly also prevent the development of exhaustion syndrome.

### Strengths and limitations

This is the first large RCT evaluating an individual or group educational intervention concerning issues relevant to middle-aged women with symptoms commonly associated with stress. The two-factor design with a high follow-up rate is the main strengths of this RCT. The contents of PCS and GE does not have its main focus on hormones and medication, but rather focus on normal aging and common psycho-social aspects of life specifically related to middle-aged women. Most of patient education recommended in PHC only have evidence for short-term follow-up, often less than 6-months. It was a strength that this study also had a long-term 12 months follow-up since it was evident that the result at 12 months was quite different from that at 6 months.

This study calculated many p-values which might be considered a weakness since some of them may reach statistical significance purely by chance. However, all inferential statistics in Table 2 for PCS at 12 months strongly point in the same direction, which would not happen if most of these results were caused by pure chance. Hence, we consider our results robust.

Menopause status was not registered at the first assessment, only at the 12-month follow-up, which is a limitation. In this study, participants were recruited by advertisement, which might be considered either a limitation or strength. However, reaching motivated women early through advertising aligns with PHC´s mission of preventive health work.

### Mental health

Women have a higher risk of mood changes during the menopause transition [1, 8]. However, presence of psychosocial factors has a much stronger association with mental health than the biological stages of menopause [1]. Improving mental health, as was done in this study by PCS, might reduce the risk of experiencing life as uncontrollable, overloaded and stressful and it may also reduce the risk for the development of CMD [10]. Since PHC treats almost 70% of the patients diagnosed with CMD [11, 17] our study´s outcome clinically relevant.

GE significantly increased depressive symptoms measured by MADRS-S at the 6 month follow-up. This negative effect vanished at the 12-month follow-up. A similar trend was seen for PCS with a weak negative effect at 6 months changing to a significantly positive effect on the mood at 12 months. One possible explanation could be that initially the interventions make women aware of their shortcomings, but they have not yet been able to address them. As time goes by, they gradually address shortcomings and become less depressed [8]*.*

### Urogenital health

Nearly 50% of middle-aged women are affected by urinary incontinence and this significantly reduces their quality of life [1, 3, 25]. Roughly half of all postmenopausal women have symptoms of local estrogen deficiency [26]. Menopausal symptoms (measured by MRS) affect 80% of women’s quality of life and 42% of women report these symptoms as very serious [8, 23, 27, 28]. MRS is a good age- and condition-specific quality of life questionnaire for use among middle-aged women to measure the severity of these symptoms and their impact on the quality of life [28]. This makes MRS useful in PHC for the assessment of the quality of life in a standardized way to guide treatment options [28].

A previous quasi-experimental study with short-term follow-up found that group education related to women’s urogenital health during menopause improved urogenital symptoms and quality of life [15]. The present study is in line with this but clarifies that the effect of GE, as provided in this present study, vanish after 6 months and only the effect of PCS lasts long-term.

### Somatic health

Menopausal vasomotor symptoms (VMS) affect 60–80% of middle-aged women and have been strongly related to reduced quality of life impacting physical, psychosocial, sleep and overall wellbeing [1, 27, 29]. For more than half of the women, VMS remains more than 7 years during the menopausal transition [28]. These problems are on the rise so it is not surprising that also cardiovascular symptoms and the incidence of cardiovascular disease (CVD) are increasing for middle aged women [6]. The present study did not have long enough follow-up to show effect on CVD. However, risk factors for CVD were discussed in the interventions and it is likely the PCS intervention may have some positive effect on that in the long run.

### Group education and person-centered individual support

Previous studies, with follow-up periods varying between six weeks and 6 months, showed that group education for middle-age women had a positive effect on health-related quality of life and menopause symptoms [15, 16]. Hence, before initiating this present study we expected GE to be the most effective treatment and that is the reason the effect of GE was made the primary research outcome. However, it turned out that any positive effect of GE vanished after 6 months and the strongest effect was, somewhat surprising, for PCS at 12 months after the completed intervention. Hence, a follow-up period longer than 6 months seems very important since the short- and long-term results differed.

## Conclusion

Person-centered individual support on topics related to menopause to women aged 45–60 improved health-related quality of life, and reduced mental, somatic, urogenital, and stress-related symptoms with an effect lasting at least 12 months. To improving women’s health these results, suggest that person-centered individual support on menopause could be an effective intervention in PHC.

## Supplementary Information

**Additional file 1: Table S1a** Changes from baseline to 6-month follow-up (n = 287). **Table S1b** Changes in health-related Quality of Life at 12-month follow-up in the four groups (n = 289). **Table S2** The effect of group education and person-centered individual support at 6-month follow-up using linear regression. **Table S3** The effect of group education and person-centered individual support at 6-month follow-up using ordinal regression. **Table S4** The effect of group education and person-centered individual support at 12-month follow-up using linear regression. **Table S5** The effect of group education and person-centered individual support at 12-month follow-up using ordinal regression.

## Data Availability

The datasets used and/or analysed during the current study available from the corresponding author on reasonable request.
