# Supplementary material for: Effect of group education and person-centered support in primary health care on mental health and quality of life in women aged 45–60 years with symptoms commonly associated with stress: a randomized controlled trial
Source: BMC Womens Health. 2023 Mar 24;23:128. doi: 10.1186/s12905-023-02221-6 (PMC10039535; doi:10.1186/s12905-023-02221-6)
Supplement: Supplementary file 1 — Additional file 1: Table S1a Changes from baseline to 6-month follow-up (n = 287). Table S1b Changes in health-related Quality of Life at 12-month follow-up in the four groups (n = 289). Table S2 The effect of group education and person-centered individual support at 6-month follow-up using linear regression. Table S3 The effect of group education and person-centered individual support at 6-month follow-up using ordinal regression. Table S4 The effect of group education and person-centered individual support at 12-month follow-up using linear regression. Table S5 The effect of group education and person-centered individual support at 12-month follow-up using ordinal regression. [file 12905_2023_2221_MOESM1_ESM.docx]

**Supplemental tables S1-S5.**

| **Table S1a. Changes from baseline to 6-month follow-up (n=287).** | | | | | | | | | | |
| --- | --- | --- | --- | --- | --- | --- | --- | --- | --- | --- |
|  | Group 1 - GE  n=64 | | Group 2 – GE+PCS  n=68 | | Group 3 - PCS  n= 81 | | Group 4 - Control  n=74 | | Total  n=287 | |
| Work status/ Employment status^a^ |  | |  | |  | |  | |  | |
| Currently working^b^ | 52 → 55 | | 62 → 59 | | 68 → 73 | | 64 → 70 | | 246 → 257 | |
| Sick leave full-time/part-time^b^ | 9 → 5 | | 3 → 7 | | 4 → 6 | | 2 → 1 | | 26 → 21 | |
| Disability pension full-time/part-time ^b^ | 0 → 4 | | 0 → 2 | | 0 → 2 | | 3 → 1 | | 3 → 9 | |
| Visit PHCC last 2 months^b,c^ | 25 → 27 | | 28 → 31 | | 41 → 36 | | 37 → 33 | | 131 → 127 | |
| Cause of recent visit PHCC^b,c^ |  | |  | |  | |  | |  | |
| Physical discomfort | 42 → 28 | | 38 → 28 | | 42 → 38 | | 40 → 38 | | 162 → 132 | |
| Psychological discomfort | 16 → 11 | | 15 → 11 | | 16 → 10 | | 9 → 11 | | 56 → 43 | |
| Menopause^b^ |  | |  | |  | |  | |  | |
| MHT (Menopause Hormone Therapy) |  | |  | |  | |  | |  | |
| MHT Treatment system tablet | 3 → 7 | | 4 → 5 | | 6 → 13 | | 5 → 10 | | 18 → 35 | |
| MHT Treatment local | 9 → 13 | | 4 → 10 | | 7 → 9 | | 4 → 2 | | 24 → 34 | |
| MHT not use | 52 → 44 | | 60 → 53 | | 68 → 59 | | 65 → 62 | | 245 → 218 | |
| MHT use | 12 → 20 | | 8 → 15 | | 13 → 22 | | 9 → 12 | | 42 → 69 | |
| AUDIT total^d,e^ | +0.27 (1.9) | 0.00 (1.0) | +0.073 (1.4) | 0.00 (1.0) | -0.31 (1.3) | 0.0 (1.0) | +0.040 (0.91) | 0.00 (0.0) | 0.0 (1.4) | 0.0 (0.0) |
| Health Related Quality of Life |  |  |  |  |  |  |  |  |  |  |
| SF-36^d,f,g^ |  |  |  |  |  |  |  |  |  |  |
| Physical Function (PF)  PP (n=280) | -0.47 (13) | 0.0 (10) | + 2.4 (20) | 0.0 (10) | + 2.4 (18) | 0.0 (5) | +2.8 (21) | 0.0 (5) | +1.9 (18) | 0.0 (5) |
| CC (n=287) | -0.41 (13) | 0.0 (10) | + 2.4 (20) | 0.0 (10) | +2.7 (18) | 0.0 (5) | +2.8 (21) | 0.0 (5) | +2.0 (19) | 0.0 (5) |
| ITT (n=368) | -0.36 (14) | 0.0 (10) | +2.8 (20) | 0.0 (10) | +2.6 (18) | 0.0 (5) | +2.5 (18) | 0.0 (5) | +1.5 (16) | 0.0 (5) |
| Role Physical (RP)  CC (n=287) | +9.0 (40) | 0.0 (25) | +9.6 (41) | 0.0 (25) | +13 (39) | 0.0 (25) | +11 (41) | 0.0 (25) | +11 (40) | 0.0 (25) |
| PP (n=280) | +9.0 (39) | 0.0 (25) | +9.9 (42) | 0.0 (31) | +14 (40) | 0.0 (25) | +11 (41) | 0.0 (25) | +11 (40) | 0.0 (25) |
| ITT (n=368) | +7.6 (40) | 0.0 (25) | +7.8 (41) | 0.0 (25) | +12 (41) | 0.0 (25) | +12 (40) | 0.0 (25) | +9.1 (36) | 0.0 (25) |
| Bodily Pain (BP)  CC (n=287) | +2.4 (24) | 0.0 (28) | +1.8 (24) | +0.50 (32) | +3.8 (22) | 0.0 (22) | +6.6 (24) | +10 (31) | +3.7 (23) | 0.0 (29) |
| PP (n=280) | +1.4 (24) | 0.0 (24) | +3.6 (21) | +1.0 (31) | +3.7 (22) | 0.0 (26) | +6.6 (24) | +10 (31) | +4.0 (23) | 0.0 (29) |
| ITT (n=368) | +2.1 (24) | 0.0 (28) | +1.9 (24) | 0.0 (34) | +3.1 (22) | 0.0 (26) | +8.7 (24) | +10 (31) | +2.9 (21) | 0.0 (12) |
| General Health (GH)  CC (n=287) | +1.7 (16) | 0.0 (17) | +8.9 (19) | +9.0 (27) | +4.0 (13) | +3.0 (15) | +4.7 (18) | +5.0 (21) | +4.8 (17) | +5.0 (20) |
| PP (n=280) | +1.5 (17) | 0.0 (18) | +9.8 (18) | +10 (28) | +4.2 (13) | +3.0 (15) | +4.7 (18) | +5.0 (21) | +5.0 (17) | +5.0 (20) |
| ITT (n=368) | +1.7 (17) | 0.0 (18) | +7.4 (18) | +5.0 (26) | +4.0 (14) | +5.0 (15) | 5.6 (18) | +5.0 (22) | +3.8 (15) | 0.0 (12) |
| Vitality (VT)  CC (n=287) | +7.0 (21) | +5.0 (25) | +15 (18) | +15 (25) | +9.4 (20) | +10 (28) | +8.6 (18) | +10 (21) | +10 (19) | 10 (30) |
| PP (n=280) | +6.7 (21) | +5.0 (28) | +15 (18) | +15 (25) | +9.5 (20) | +10 (30) | +8.6 (18) | +10 (21) | +10 (20) | 10 (30) |
| ITT (n=368) | +7.0 (22) | +5.0 (25) | +15 (18) | +15 (25) | +10 (20) | +10 (30) | +8.7 (19) | +10 (20) | +7.8 (18) | 0.0 (20) |
| Social function (SF)  CC (n=287) | +6.8 (24) | 0.0 (34) | +12 (25) | +13 (25) | +11 (24) | 0.0 (25) | +4.7 (20) | 0.0 (13) | +8.6 (23) | 0.0 (25) |
| PP (n=280) | +7.2 (25) | 0.0 (25) | +12 (25) | +13 (25) | +11 (24) | +13 (25) | +4.7 (20) | 0.0 (13) | +8.8 (24) | 13 (25) |
| ITT (n=368) | +8.4 (25) | 0.0 (25) | +12 (25) | +13 (25) | +12 (25) | +13 (25) | +4.2 (20) | 0.0 (13) | +6.7 (21) | 0.0 (13) |
| Role Emotional RE  CC (n=287) | +10 (44) | 0.0 (33) | + 24 (41) | +33 (33) | +13 (46) | 0.0 (33) | +10 (41) | 0.0 (33) | +14 (44) | 0.0 (33) |
| PP (n=280) | +9.8 (45) | 0.0 (33) | +23 (41) | +33 (33) | +14 (47) | 0.0 (33) | +10 (41) | 0.0 (33) | +14 (44) | 0.0 (33) |
| ITT (n=368) | +11 (46) | 0.0 (33) | +23 (41) | +33 (33) | +16 (44) | 0.0 (33) | +9.0 (44) | 0.0 (33) | +11 (39) | 0.0 (33) |
| Mental Health (MH)  CC (n=287) | +9.4 (21) | +8.0 (24) | +11 (18) | +8.0 (20) | +7.5 (19) | +4.0 (22) | +1.1 (18) | 0.0 (20) | +7.2 (19) | +4.0 (20) |
| PP (n=280) | +9.8 (21) | +8.0 (24) | +12 (18) | +8.0 (21) | +7.8 (19) | +8.0 (20) | +1.1 (18) | 0.0 (20) | +7.4 (20) | +4.0 (20) |
| ITT (n=368) | +11 (22) | +10 (24) | +10 (17) | +8.0 (18) | +9.4 (18) | 8.0 (20) | +1.9 (18) | 0.0 (24) | +5.6 (17) | 0.0 (12) |
| PCS  CC (n=287) | -0.35 (8.9) | -0.35 (10) | +0.072 (9.8) | +1.6 (10) | +1.3 (9.2) | +0.13 (9) | +2.5 (10) | +1.5 (8) | +0.96 (9.6) | +0.37 (22) |
| PP (n=280 | -0.48 (8.9) | -0.29 (10) | +0.33 (9.8) | +1.9 (10) | +1.3 (9.3) | -0.27 (10) | +2.5 (10) | +1.5 (8) | +1.0 (9.7) | +0.39 (10) |
| ITT (n=368) | -0.68 (9.2) | -0.63 (11) | +0.050 (10) | +1.8 (10) | +0.64 (8.7) | -0.34 (10) | +2.9 (10) | +2.1 (7) | +0.74 (8.5) | +0.010 (7) |
| MCS  CC 287 | +5.3 (13) | +3.7 (16) | +8.9 (13) | +7.0 (16) | +5.4 (13) | +2.7 (16) | +2.3 (11) | +1.5 (15) | +5.4 (13) | +3.2 (15) |
| PP (280) | +5.3 (13) | +3.6 (17) | +9.1 (12) | +7.0 (16) | +5.5 (13) | +2.6 (17) | +2.3 (12) | +1.5 (15) | +5.5 (13) | +3.6 (15) |
| ITT (n=368) | +5.9 (13) | +4.5 (18) | +8.6 (12) | +7.0 (16) | +6.7 (12) | +2.7 (17) | +2.2 (12) | +1.1 (16) | +4.2 (12) | +0.46 (10) |
| HADS^d,g,h^ |  |  |  |  |  |  |  |  |  |  |
| Depression  PP (n=280) | -1.0 (4.8) | -1.5 (6) | -1.6 (5.6) | -1.0 (7) | 0.73 (5.1) | 0.0 (7) | -1.7 (5.3) | -2.0 (9) | -1.2 (5.2) | -1.0 (7) |
| CC (n=287) | -0.88 (4.8) | 0.0 (6) | -1.5 (5.7) | -1.0 (8) | -0.82 (5.1) | 0.0 (7) | -1.6 (5.4) | -2.0 (9) | -1.2 (5.2) | 1.0 (7) |
| ITT (n=368) | -0.86 (4.8) | 0.0 (6) | -1.0 (5.3) | -1.0 (8) | -0.55 (5.1) | 0.0 (7) | -1.5 (5.4) | -2.0 (9) | -0.93 (4.6) | 0.0 (5) |
| Anxiety  PP (n=280) | -1.1 (6.4) | -1.0 (9) | -1.9 (6.1) | -1.0 (9) | -1.7 (6.3) | -3.0 (8) | -1.8 (6.5) | -2.0 (9) | -1.6 (6.3) | -2.0 (9) |
| CC (n=287) | 1.1 (6.3) | -1.0 (9) | 1.8 (6.1) | -1.0 (9) | -1.8 (6.4) | -3.0 (8) | -1.8 (6.5) | -2.0 (9) | -1.6 (6.3) | -2.0 (9) |
| ITT (n=368) | -1.0 (6.5) | -1.0 (9) | -1.2 (5.8) | 0.0 (8) | -1.2 (6.2) | -2.0 (8) | -1.6 (6.2) | -2.0 (9) | -1.3 (5.6) | 0.0 (5) |
| s-ED^d,g,i^  CC (n=287) | -0.82 (2.0) | 0.0 (2) | -1.0 (1.6) | -0.14 (2) | -1.1 (1.6) | -0.15 (2) | -0.55 (2.0) | 0.0 (1) | -0.87 (1.8) | 0.0 (2) |
| PP (n=280) | -0.89 (2.0) | 0.15 (2) | -1.0 (1.6) | -0.15 (2) | -1.2 (1.6) | -0.15 (2) | -0.55 (2.0) | 0.0 (1) | -0.90 (1.8) | -0.15 (2) |
| ITT (n=368) | -0.82 (2.1) | 0.0 (2) | -1.0 (1.7) | 0.0 (2) | -1.2 (1.7) | 0.0 (2) | -0.57 (2.1) | 0.0 (2) | -0.71 (1.7) | 0.0 (2) |
| PSS-14^d,g,j^  CC (n=287) | -2.3 (8.9) | -1.0 (11) | -4.3 (9.6) | -4.0 (14) | -1.9 (7.3) | -1.0 (11) | -0.66 (7.3) | -1.0 (9) | -2.2 (8.3) | -2.0 (10) |
| PP n= (280) | -2.3 (9.0) | -1.0 (11) | -4.8 (9.3) | -4.5 (13) | -2.0 (7.4) | -1.0 (11) | -0.66 (7.3) | -1.0 (9) | -2.4 (8.3) | -2.0 (10) |
| ITT(n=368) | -2.6 (9.3) | -1.5 (12) | -4.0 (9.2) | -4.0 (13) | -2.3 (7.6) | -1.0 (11) | -0.83 (7.7) | -1.0 (9) | -2.5 (7.4) | 0.0 (8) |
| MADRS-S^d,g,k^  CC (n=287) | -3.6 (6.9) | -4.0 (9) | -5.9 (8.0) | -4.0 (9) | -4.2 (6.3) | -3.0 (10) | -4.5 (4.6) | -4.0 (5) | -4.5 (6.5) | -4.0 (8) |
| PP (n=280) | -3.5 (7.1) | -4.0 (9) | -6.1 (8.2) | -4.0 (9) | -4.2 (6.4) | -3.0 (10) | -4.5 (4.6) | -4.0 (5) | -4.6 (6.6) | -4.0 (8) |
| ITT (n=368) | -3.9 (7.0) | -4.0 (9) | -5.8 (7.8) | -4.0 (8) | -4.7 (6.3) | -3.0 (10) | -4.8 (4.9) | -4.0 (6) | -3.5 (6.1) | -2.0 (6) |
| MRS^d,g,l^ |  |  |  |  |  |  |  |  |  |  |
| Somatic  CC (n=287) | -1.3 (2.2) | -1.0 (3) | -1.2 (2.6) | -1.0 (3) | -0.78 (2.6) | -1.0 (3) | -0.54 (2.6) | 0.0 (3) | -0.93 (2.5) | -1.0 (3) |
| PP (n=280) | -1.3 (2.2) | -1.0 (3) | -1.2 (2.6) | -1.0 (3) | -0.75 (2.6) | -1.0 (3) | -0.54 (2.6) | 0.0 (3) | -0.91 (2.5) | -1.0 (3) |
| ITT (n=368) | -1.4 (2.3) | -1.0 (3) | -1.1 (2.5) | -1.0 (2) | -0.85 (2.6) | -1.0 (3) | -0.95 (2.5) | 0.0 (3) | -0.73 (2.3) | 0.0 (2) |
| Urogenital  CC (n=287) | -0.69 (2.3) | 0.0 (3) | -1.1 (2.4) | -1.0 (3) | -0.84 (2.1) | 0.0 (2) | 0.0 (1.8) | 0.0 (2) | -0.63 (2.2) | 0.0 (3) |
| PP (n=280) | -0.75 (2.3) | 0.0 (3) | -0.97 (2.4) | -1.0 (3) | -0.76 (2.0) | 0.0 (2) | 0.0 (1.8) | 0.0 (2) | -0.61 (2.1) | 0.0 (3) |
| ITT (n=368) | -0.62 (2.4) | 0.0 (3) | -0.86 (2.3) | -1.0 (2.5) | -0.84 (2.2) | 0.0 (2) | -0.14 (1.9) | 0.0 (2) | -0.49 (1.9) | 0.0 (1) |
| Psychological  CC (n=287) | -1.7 (3.2) | -2.0 (4) | -2.7 (3.5) | -2.0 (5) | -1.5 (2.8) | -1.0 (3) | -0.55 (3.1) | 0.0 (3) | -1.6 (3.2) | -1.0 (3) |
| PP (n=280) | -1.7 (3.2) | -2.0 (4) | -2.7 (3.6) | -2.0 (5) | -1.5 (2.9) | -1.0 (3) | -0.55 (3.1) | 0.0 (3) | -1.6 (3.2) | -1.0 (3) |
| ITT (n=368) | -1.8 (3.3) | -1.5 (5) | -2.5 (3.2) | -2.0 (4) | -1.7 (2.8) | -1.0 (3) | -0.76 (3.2) | 0.0 (2) | -1.2 (2.9) | 0.0 (3) |
| Total  CC (n=287) | -3.7 (6.1) | -3.0 (6) | -4.8 (6.9) | -4.0 (7) | -3.1 (5.7) | -2.0 (6) | -1.1 (5.7) | 0.0 (7) | -3.1 (6.2) | -2.0 (7) |
| PP (n=280) | -3.8 (6.2) | -3.0 (6) | -4.9 (7.0) | -4.0 (7) | -3.1 (5.6) | -2.0 (5) | -1.1 (5.7) | 0.0 (7) | -3.1 (6.2) | -2.0 (7) |
| ITT (n=368) | -3.8 (6.5) | -3.0 (6) | -4.4 (6.4) | -4.0 (7) | -3.4 (5.8) | -2.0 (6) | -1.9 (5.8) | -1.0 (8) | -2.4 (5.6) | 0.0 (5) |
| ^a^ Work status, paid work more than one hour/week.  ^b^ First figure number of patients reporting in baseline currently working, sick leave, disability pension, visit PHC and Menopause Hormone Therapy (MHT). Second figure number reporting at 12 months follow up.  ^c^ Primary Health Care Center (PHCC) ^d^ First figure changes in Mean (SD), second figure changes in Median (interquartile range).  ^e^ Changes in measure in AUDIT –alcohol habits, identify risky use, harmful use or alcohol dependence, Negative values indicate decrease in alcohol consumption.  ^f^ Changes in measure in quality of life, SF-36. Physical component score (PCS) and mental component score (MCS) separately along with eight domains of SF-36. Positive value increases reflect a better Health Related Quality of Life.  ^g^ First line Per Protocol, second line Complete Case (CC), third line Intention-to-treat (ITT). Per Protocol, received allocated intervention and responded to follow-up survey. Complete Case; responded to follow-up survey. Intention To Treat all participants included with last outcome carried forward (LOCF).  ^h^ Changes in measure in Hospital Anxiety and Depression scale (HADS). Negative values indicate decreased anxiety and depression.  ^i^ Changes in measure in Self-rated Exhaustion Disorder (s-ED) identifies risk to develop exhaustion disorder with reduced workability and increased risk of sick leave. Negative values indicate decreased risk.  ^j^ Changes in measure in Perceived Stress Scale 14 (PSS-14). Negative values indicate decrease of mental stress. ^k^ Changes in measure in Montgomery-Asberg Depression Rating Scale (MADRS-S) scoring. Negative values indicate decrease in depression. ^l^ Changes in measure in Menopause Rating Scale (MRS) measure prevalence and severity of aging signs and Health Related Quality of Life. MRS subscale: Somatic symptoms - hot flushes, heart discomfort, sleeping problems and muscle and joint problems, Psychological symptoms - depressive mood, irritability, anxiety and physical and mental exhaustion, Urogenital symptoms - sexual problems, bladder problems and vaginal dryness, Total score - all subscales added. Negative values indicate improved Health Related Quality of Life. | | | | | | | | | | |

| **Table S1b. Changes in health-related Quality of Life at 12-month follow-up in the four groups (n=289).** | | | | | | | | | | |
| --- | --- | --- | --- | --- | --- | --- | --- | --- | --- | --- |
|  | Group 1 - GE  n=64 | | Group 2 – GE+PCS  n=69 | | Group 3 - PCS  n= 81 | | Group 4^d^- Control  n=75 | | Total  n=289 | |
|  |  | |  | |  | |  | |  | |
| Work status/ Employment status^a,b^ |  | |  | |  | |  | |  | |
| Currently working | 53 → 55 | | 62 → 60 | | 71 → 71 | | 66 → 71 | | 252 → 257 | |
| Sick leave full-time/part-time | 9 → 5 | | 3 → 7 | | 6 → 5 | | 5 → 1 | | 23→ 18 | |
| Disability pension full-time/part-time | 2 → 3 | | 4 → 1 | | 4 → 3 | | 2 → 2 | | 12 → 9 | |
| Visit PHCC last 2 months^b,c^ | 27 → 25 | | 42 → 33 | | 40 → 43 | | 37 → 37 | | 156 →138 | |
| Cause of visit PHCC^c^ |  | |  | |  | |  | |  | |
| Physical discomfort | 41 → 35 | | 40 → 26 | | 40 → 29 | | 40 → 29 | | 161 → 119 | |
| Psychological discomfort | 14 → 7 | | 17 → 9 | | 16 → 9 | | 9 → 6 | | 56 → 31 | |
| Menopause status^d,e^ |  | |  | |  | |  | |  | |
| I have regular menstruation | 5 (8) | | 6 (9) | | 8 (10) | | 17 (22) | | 36 (12) | |
| I have irregular bleeding, within the last 12 months | 9 (15) | | 14 (21) | | 13 (16) | | 16 (21) | | 52 (18) | |
| I have not had a period in > 12 months | 33 (52) | | 39 (57) | | 43 (53) | | 27 (36) | | 142 (49) | |
| I have IUDs or other hormonal contraceptives that affect my bleeding | 17 (27) | | 9 (13) | | 12 (15) | | 14 (18) | | 52 (18) | |
| MHT   (Menopause Hormone Therapy)^b^ |  | |  | |  | |  | |  | |
| MHT Treatment system tablet | 4 → 10 | | 5 → 7 | | 6 → 15 | | 5 → 11 | | 20 → 43 | |
| MHT Treatment local | 9 → 11 | | 5 → 10 | | 6 → 13 | | 5 → 3 | | 25 → 37 | |
| MHT not use | 51 → 43 | | 59 → 52 | | 69 → 53 | | 65 → 61 | | 244 → 209 | |
| MHT use | 13 → 21 | | 10 → 17 | | 12 → 28 | | 10 → 14 | | 45 → 80 | |
| AUDIT^f,g^ | +0.13 (2.7) | 0.0 (2.0) | +0.06 (1.3) | 0.0 (1.0) | +0.68 (1.6) | 0.0 (2.0) | +0.29 (1.2) | 0.0 (0.0) | -3.4 (8.1) | -3.0 (10) |
| Health related Quality of Life |  |  |  |  |  |  |  |  |  |  |
| SF-36^f,h^ |  |  |  |  |  |  |  |  |  |  |
| Physical Function (PF)  PP (n=284) | -0.74 (14) | 0.0 (10) | + 3.3 (16) | 0.0 (10) | + 3.1 (17) | 0.0 (9) | +2.8 (18) | 0.0 (10) | +2.2 (17) | 0.0 (10) |
| CC (n=289) | -0.31 (15) | 0.0 (10) | +3.1 (16) | 0.0 (13) | +3.0 (17) | 0.0 (8) | +2.6 (18) | 0.0 (10) | +2.3 (17) | 0.0 (10) |
| ITT (n=368) | - 0.11 (12) | 0.0 (5) | +2.8 (14) | 0.0 (5) | +3.9 (18) | 0.0 (8) | +1.8 (17) | 0.0 (5) | +2.2 (17) | 0.0 (10) |
| Role Physical (RP)  PP (n=284) | +3.7 (48) | 0.0 (25) | +13 (36) | 0.0 (25) | +17 (41) | 0.0 (25) | +8.3 (42) | 0.0 (25)) | +11 (41) | 0.0 (25) |
| CC (n=289) | +4.3 (46) | 0.0 (25) | +3.7 (36) | 0.0 (20) 5 | +17 (41) | 0.0 (25) | +8.7 (42) | 0.0 (25) | +11 (42) | 0.0 (25) |
| ITT (n=368) | +6.6 (42) | 0.0 (25) | +9.1 (32) | 0.0 (25) | +16 (40) | 0.0 (25) | +7.5 (40) | 0.0 (25 | +11 (41) | 0.0 (25) |
| Bodily Pain (BP)  PP (n=280) | -4.3 (27) | 0.0 (30) | +6.8 (23) | 0.0 (26) | +8.6 (24) | +10 (22) | +12 (71) | +10 (29) | +3.7 (25) | 0.0 (30) |
| CC (n=289) | -4.1 (27) | 0.0 (30) | +6.7 (23) | 0.0 (26) | +8.2 (24) | +10 (22) | +3.0 (25) | +10 (30) | +4.1 (25) | 0.0 (30) |
| ITT (n=368) | -2.5 (23) | 0.0 (10) | +3.9 (23) | +5.0 (12) | +8.2 (24) | +10 (22) | +7.9 (64) | 0.0 (26) | +6.1 (42) | 0.0 (29) |
| General Health (GH)  PP (n=284) | +0.52 (17) | 0.0 (20) | +6.0 (17) | +5.0 (22) | +7.5 (16) | +10 (19) | +4.9 (19) | +5.0 (20) | +4.8 (17) | +5.0 (20) |
| CC (n=289) | +0.81 (17) | 0.0 (20) | +5.8 (17) | +5.0 (21) | +7.5 (16) | +10 (19) | +4.4 (19) | +5.0 (20) | +5.0 (17) | +5.0 (20) |
| ITT (n=368) | +0.79 (14) | 0.0 (10) | +4.6 (15) | 0.0 (15) | +7.2 (15) | +5.0 (15) | +3.7 (18) | 0.0 (20) | +5.0 (17) | +5.0 (20) |
| Vitality (VT)  PP (n=284) | +7.1 (22) | +5.0 (20) | +14 (22) | +15 (34) | +12 (20) | +10 (25) | +5.8 (19) | +5.0 (20) | +9.3 (21) | +10 (25) |
| CC (n=289) | +6.1 (22) | +5.0 (24) | +13 (22) | +15 (35) | +12 (20) | +10 (25) | +5.8 (19) | +5.0 (20) | +9.7 (21) | +10 (25) |
| ITT (n=368) | +5.3 (19) | 0.0 (15) | +11 (21) | +5.0 (25) | +11 (19) | +10 (23) | +4.8 (18) | 0.0 (20) | +9.6 (21) | +10 (25) |
| Social function (SF)  PP (n=284) | +11 (24) | 0.0 (25) | +14 (24) | +13 (25) | +11 (25) | +13 (25) | +1.2 (24) | 0.0 (25) | +8.7 (25) | 0.0 (25) |
| CC (n=289) | +9.4 (24) | 0.0 (25) | +14 (24) | +13 (25) | +10 (25) | +13 (25) | +1.3 (24) | 0.0 (25) | +9.1 (25) | 0.0 (25) |
| ITT (n=368) | +7.3 (21) | 0.0 (13) | +11 (24) | 0.0 (25) | +9.4 (25) | +0.50 (25) | +2.1 (22) | 0.0 (13) | +9.0 (25) | 0.0 (25) |
| Role Emotional RE  PP (n=284) | +9.8 (46) | 0.0 (33) | + 22 (44) | +0.0 (58) | +16 (42) | 0.0 (33) | +6.7 (46) | 0.0 (33) | +14 (45) | 0.0 (33) |
| CC (n=289) | +8.3 (49) | 0.0 (33) | +22 (44) | 0.0 (50) | +16 (42) | 0.0 (33) | +8.0 (46) | 0.0 (33) | +14 (45) | 0.0 (33) |
| ITT (n=368) | +8.1 (42) | 0.0 (33) | +18 (40) | 0.0 (33) | +15 (41) | 0.0 (33) | +7.2 (43) | 0.0 (0) | +14 (45) | 0.0 (33) |
| Mental Health (MH)  PP (n=284) | +7.6 (22) | +4.0 (24) | +11 (21) | +8.0 (24) | +10 (16) | +8.0 (19) | +1.7 (18) | 0.0 (16) | +9.9 (19) | +4.0 (20) |
| CC (n=289) | +5.1 (22) | 0.0 (24) | +11 (21) | +8.0 (24) | +10 (16) | +8.0 (19) | +1.3 (18) | 0.0 (16) | +7.5 (20) | +4.0 (20) |
| ITT (n=368) | +4.5 (20) | 0.0 (16) | +9.3 (19) | +4.0 (20) | +10 (16) | +8.0 (18 | +1.3 (17) | 0.0 (12) | +7.3 (19) | +4.0 (20) |
| PCS  PP (n=284) | -1.7 (11) | -1.1 (11) | +0.66 (8.9) | -0.44 (9) | +2.2 (9.0) | +2.4 (9) | +1.9 (9.7) | +1.3 (12) | +0.97 (9.7) | 1.1 (11) |
| CC (n=289) | -1.2 (11) | -0.98 (11) | +0.96 (8.6) | +0.50 (9) | +2.2 (9.0) | +2.4 (9) | +1.9 (9.9) | +1.3 (13) | +1.1 (9.6) | +1.1 (11) |
| ITT (n=368) | - 0.72 (9.3) | - 0.13 (8) | +0.26 (7.9) | -0.17 (7) | +2.4 (8.9) | +1.6 (9) | +1.1 (9.2) | +0.29 (10) | +0.78 (8.9) | +0.17 (8) |
| MCS  PP (n=284) | +6.0 (13) | +2.5 (20) | +8.4 (14) | +6.7 (17) | +6.4 (12) | +5.2 (12) | +1.5 (12) | +0.70 (16) | +5.2 (13) | +4.0 (15) |
| CC (n=289) | +4.7 (14) | +2.3 (19) | +8.3 (14) | +6.2 (17) | +6.4 (12) | +5.2 (12) | +15 (12) | +0.70 (16) | +5.4 (13) | +4.0 (15) |
| ITT (n=368) | +3.9 (12) | +0.28 (11) | +6.5 (13) | +13 (13) | +5.9 (12) | +4.0 (11) | +1.80 (11) | +0.35 (14) | +5.4 (13) | +4.4 (15) |
| HADS^f,i^ |  |  |  |  |  |  |  |  |  |  |
| Depression  PP (n=284) | -1.0 (4.8) | -1.0 (6) | -1.8 (5.4) | -1.0 (7) | -1.2 (5.2) | -1.0 (8) | -1.6 (5.4) | -3.0 (9) | -1.4 (5.1) | -1.0 (7) |
| CC (n=289) | -1.0 (4.6) | -1.0 (6) | -1.6 (5.4) | -1.0 (7) | -1.3 (5.2) | -1.0 (8) | -1.6 (5.4) | -3.0 (9) | -1.5 (5.2) | -1.0 (7) |
| ITT (n=368) | -0.71 (4.1) | 0.0 (4) | -1.4 (4.8) | 0.0 (5) | -0.96 (5.03) | -1.0 (7) | -1.3 (5.01) | 0.0 (9) | -1.4 (5.2) | -1.0 (7) |
| Anxiety  PP (n=284) | -0.66 (6.4) | -0.50 (8) | -2.8 (5.9) | -2.0 (7) | -3.0 (6.3) | -4.0 (8) | -1.2 (6.4) | -1.0 (9.0) | -1.9 (6.2) | -2.0 (8) |
| CC (n=289) | -0.70 (6.3) | -0.50 (8) | -2.8 (5.6) | -2.0 (7) | -2.6 (6.4) | -4.0 (9) | -1.4 (6.3) | -1.0 (8) | -2.0 (6.2) | -2.0 (8) |
| ITT (n=368) | -0.49 (5.4) | 0.0 (5) | -2.2 (5.1) | -1.0 (5) | -1.0 (5) | -2.0 (8) | -1.1 (5.9) | 0.0 (7) | -1.9 (6.2) | -2.0 (8) |
| s-ED^f,j^  PP (n=284) | -1.7 (2.8) | -2.0 (3) | -1.9 (3.0) | -2.0 (4) | -2.1 (3.0) | -2.2 (4) | -1.6 (3.0) | -2.0 (4) | -1.8 (3.0) | -2.0 (4) |
| CC (n=289) | -1.7 (2.9) | -2.0 (3) | -1.9 (2.9) | -2.0 (4) | -2.1 (3.0) | -2.0 (4) | -1.6 (3.0) | -2.0 (4) | -1.8 (2.9) | -2.0 (4) |
| ITT (n=368) | -1.2 (2.6) | -1.0 (3) | -1.5 (2.6) | -1.0 (4) | -1.8 (2.9) | -1.1 (4) | -1.3 (2.8) | -0.15 (3) | -1.8 (2.9) | -2.0 (4) |
| PSS-14^f,k^  PP (n=284) | -2.8 (9.6) | -2.0 (12) | -5.5 (7.6) | -5.0 (10) | -4.7 (7.6) | -3.5 (12) | -0.51 (7.1) | +1.0 (9) | -3.2 (8.1) | -2.0 (11) |
| CC (n=289) | -2.4 (9.4) | -1.5 (11) | -5.3 (7.7) | -5.0 (10) | -4.7 (7.6) | -3.5 (12) | -0.34 (7.1) | +1.0 (9) | -3.3 (8.1) | -2.0 (10) |
| ITT (n=368) | -2.07 (8.2) | 0.0 (7) | -3.9 (7.5) | -2.0 (8) | -4.2 (7.7) | -3.0 (10) | -0.29 (6.4) | 0.0 (8) | -2.6 (7.6) | 0.0 (8) |
| MADRS-S^f,l^  PP (n=284) | -1.4 (7.7) | ´  0.0 (8) | -3.5 (7.4) | -2.0 (9) | -2.3 (5.6) | -1.0 (7) | +0.21 (5.9) | 0.0 (7) | -1.7 (6.7) | -1.0 (7) |
| CC (n=289) | -1.2 (7.6) | 0.0 (8) | -3.5 (7.4) | -2.0 (10) | -2.9 (5.9) | -2.0 (8) | -0.64 (5.9) | -0.0 (8) | -1.7 (6.7) | -1.0 (7) |
| ITT (n=368) | -0.85 (6.3) | 0.00 (4) | -2.6 (6.6) | 0.00 (6) | -2.02 (5.3) | 0.00 (5) | -0.28 (5.4) | 0.0 (6) | -1.7 (6.7) | -1.0 (7) |
| MRS^f,m^ |  |  |  |  |  |  |  |  |  |  |
| Somatic  PP (n=284) | -1.1 (2.9) | -1.0 (5) | -1.1 (2.3) | -1.0 (4) | -1.1 (2.4) | 0.0 (2) | -0.21 (2.5) | 0.0 (3) | -0.83 (2.5) | 0.0 (3) |
| CC (n=289) | -0.91 (2.8) | -1.0 (5) | -1.0 (2.3) | -1.0 (4) | -1.1 (2.4) | -.50 (2) | -0.29 (2.6) | 0.0 (3) | -0.83 (2.5) | -1.0 (3) |
| ITT (n=368) | -0.80 (2.5) | 0.0 (3) | -0.77 (2.1) | 0.0 (2) | -1.0 (2.3) | 0.0 (2) | -0.72 (2.3) | 0.0 (2) | -0.85 (2.5) | -1.0 (3) |
| Urogenital  PP (n=284) | -0.33 (2.2) | 0.0 (3) | -0.47 (2.1) | -0.50 (2) | -0.60 (2.3) | -1.0 (2) | +0.41 (2.1) | 0.0 (2) | -0.37 (2.2) | 0.0 (2) |
| CC (n=289) | -0.20 (2.3) | 0.0 (2) | -0.46 (2.1) | 0.0 (2) | -0.79 (2.4) | -1.0 (2) | +0.040 (2.1) | 0.0 (2) | -0.36 (2.3) | 0.0 (2) |
| ITT (n=368) | +0.53 (3.1) | 0.0 (3) | +0.29 (2.4) | 0.0 (3) | -0.62 (2.7) | -1.0 (2) | -0.050 (2.4) | 0.0 (2) | -0.36 (2.2) | 0.0 (2) |
| Psychological  PP (n=284) | -1.3 (3.1) | -1.0 (4) | -2.4 (3.7) | -2.0 (5) | -2.0 (2.7) | -2.0 (4) | -0.52 (3.2) | 0.0 (3) | -1.5 (3.3) | -1.0 (3) |
| CC (n=289) | -1.1 (3.1) | -1.0 (4) | -2.4 (3.7) | -2.0 (5) | -2.0 (2.7) | -2.0 (4) | -0.56 (3.3) | 0.0 (3) | -1.5 (3.3) | -1.0 (3) |
| ITT (n=368) | -0.97 (2.9) | 0.0 (2) | -1.9 (3.4) | -1.0 (4) | -1.8 (2.7) | -1.0 (3) | -0.42 (3.0) | 0.0 (3) | -1.5 (3.3) | -0.50 (3) |
| Total  PP (n=284) | -2.7 (6.7) | -1.0 (9) | -3.9 (6.2) | -3.0 (9) | -3.7 (5.7) | -3.0 (6) | -0.67 (6.1) | 0.0 (7) | -2.7 (6.3) | -2.0 (7) |
| CC (n=289) | -2.2 (6.7) | -1.0 (8) | -3.9 (6.2) | -4.0 (9) | -3.9 (5.7) | -3.0 (7) | -0.85 (6.3) | 0.0 (7) | -2.7 (6.2) | -2.0 (8) |
| ITT (n=368) | -2.0 (6.2) | 0.0 (5) | -3.1 (5.7) | -1.0 (6) | -3.6 (5.6) | -3.0 (6) | -0.61 (5.7) | 0.0 (5) | -2.8 (6.3) | -2.0 (7) |
| ^a^ Work status, paid work more than one hour/week.  ^b^ Changes in reporting pattern from baseline to 12 months follow-up. First figure number of reporting in baseline currently working, sick leave, disability pension, visit PHC and Menopause Hormone Therapy (MHT). Second figure number reporting at 12 months follow up.  ^c^ Primary Health Care Center (PHCC)  ^d^ Menopausal sign and bleeding pattern, changes in menstruation.  ^e^ Number (percent) ^f^ First figure changes in Mean (SD), second figure changes in Median (interquartile range).  ^g^ Changes in measure in AUDIT –alcohol habits, identify risky use, harmful use or alcohol dependence, Positive value indicate decreased alcohol consumption. Score > 6 points indicated risk use. ^h^ Changes in measure in quality of life, SF-36. Physical component score (PCS) and mental component score (MCS) separately along with eight domains of SF-36. Positive value indicated better Health Related Quality of Life.  ^i^ Changes in measure in Hospital Anxiety and Depression scale (HADS). Negative values indicate increased in anxiety and depression reporting.  ^j^ Changes in measure in Self-rated Exhaustion Disorder (s-ED) identify risk that or may be about to develop a clinical fatigue syndrome with reduced ability to work and increased risk of sick leave. Negative values indicate increase in Quality of Life. ^k^ Changes in measure in Perceived Stress Scale 14 (PSS-14) - measures of mental stress, the degree to which one experiences one's life as unpredictable, uncontrollable, and overloaded. Negative values indicate increase in decrease of mental stress. ^l^ Changes in measure in Montgomery-Asberg Depression Rating Scale (MADRS-S) scoring. Negative values indicate decrease in depression.  ^m^ Changes in measure in Menopause Rating Scale (MRS) measure prevalence and severity of aging signs and Health Related Quality of Life. MRS subscale: Somatic symptoms - hot flushes, heart discomfort, sleeping problems and muscle and joint problems, Psychological symptoms - depressive mood, irritability, anxiety and physical and mental exhaustion, Urogenital symptoms - sexual problems, bladder problems and vaginal dryness, Total score - all subscales added. Negative values indicate improved Health Related Quality of Life. | | | | | | | | | | |

| **Table S2. The effect of group education and person-centered individual support at 6-month follow-up using linear regression.** | | | | | | | | |
| --- | --- | --- | --- | --- | --- | --- | --- | --- |
|  | Group education (GE) | | Person-centered Individual support (PCS) | | Interaction between  GS and PCS | | Age (years) | |
|  | β^a^ | p-value | β^a^ | p-value | β^a^ | p-value | β^a^ | p-value |
| SF-36^b^ |  |  |  |  |  |  |  |  |
| Physical Function (PF)  PP^c^ (n=280)  CC^d^ (n=287) ITT^e^ (n=368) |  |  |  |  |  |  |  |  |
|  | -0.067 | 0.16 | -0.022 | 0.62 | 0.13 | **0.048** | -0.00051 | 0.90 |
|  | -0.063 | 0.19 | 0.024 | 0.59 | 0.13 | **0.050** | 0.00021 | 0.96 |
|  | -0.061 | 0.12 | -0.016 | 0.68 | 0.11 | 0.058 | -0.0066 | 0.85 |
| Role Physical (RP)  PP (n=280)  CC (n=287)  ITT (n=368) |  |  |  |  |  |  |  |  |
|  | 4.8 | 1.0 | 0.042 | 0.34 | -0.013 | 0.84 | 0.0038 | 0.34 |
|  | -0.00063 | 0.99 | 0.040 | 0.36 | -0.013 | 0.84 | -0.0033 | 0.40 |
|  | -0.0024 | 0.95 | 0.40 | 0.29 | -0.016 | 0.76 | -0.0037 | 0.27 |
| Bodily Pain (BP)  PP (n=280)  CC(n=287)  ITT (n=368) |  |  |  |  |  |  |  |  |
|  | -0.067 | 0.18 | -0.043 | 0.35 | 0.089 | 0.20 | -0.0012 | 0.77 |
|  | -0.053 | 0.28 | -0.042 | 0.37 | 0.059 | 0.39 | 0.00021 | 0.96 |
|  | -0.051 | 0.22 | -0.031 | 0.45 | 0.050 | 0.39 | -0.0013 | 0.70 |
| General Health (GH)  PP (n=280)  CC (n=287)  ITT (n=368) |  |  |  |  |  |  |  |  |
|  | -0.062 | 0.22 | -0.018 | 0.71 | 0.15 | **0.032** | 0.0019 | 0.64 |
|  | -0.061 | 0.21 | -0.019 | 0.69 | 0.13 | **0.051** | 0.0027 | 0.50 |
|  | -0.056 | 0.18 | 0.00044 | 0.99 | 0.088 | 0.14 | 0.0011 | 0.75 |
| Vitality (VT)  PP (n=280)  CC (n=287)  ITT (n=368) |  |  |  |  |  |  |  |  |
|  | -0.059 | 0.23 | 0.0027 | 0.95 | 0.14 | **0.049** | 0.0038 | 0.35 |
|  | -0.054 | 0.27 | -0.0019 | 0.97 | 0.13 | **0.049** | 0.0035 | 0.39 |
|  | -0.063 | 0.13 | 0.0072 | 0.086 | 0.11 | 0.074 | 0.00092 | 0.80 |
| Social function (SF)  PP (n=280)  CC (n=287)  ITT (n=368) |  |  |  |  |  |  |  |  |
|  | 0.012 | 0.81 | 0.048 | 0.30 | 0.010 | 0.89 | 0.0028 | 0.49 |
|  | 0.0057 | 0.91 | 0.043 | 0.35 | 0.017 | 0.80 | 0.0030 | 0.45 |
|  | -0.015 | 0.71 | 0.043 | 0.30 | 0.017 | 0.77 | 0.0020 | 0.58 |
| Role Emotional RE  PP (n=280)  CC (n=287)  ITT (n=368) |  |  |  |  |  |  |  |  |
|  | 0.012 | 0.79 | 0.016 | 0.72 | 0.078 | 0.23 | 0.0092 | **0.018** |
|  | 0.015 | 0.74 | 0.014 | 0.75 | 0.086 | 0.17 | 0.10 | **0.011** |
|  | 0.020 | 0.61 | 0.033 | 0.40 | 0.040 | 0.48 | 0.0062 | 0.073 |
| Mental Health (MH)  PP (n=280)  CC (n=287)  ITT (n=368) |  |  |  |  |  |  |  |  |
|  | 0.12 | **0.015** | 0.11 | **0.013** | -0077 | 0.26 | 0.0063 | 0.12 |
|  | 0.12 | **0.016** | 0.11 | **0.018** | -0.080 | 0.24 | 0.0066 | 0.10 |
|  | 0.078 | 0.061 | 0.11 | **0.010** | -0.066 | 0.26 | 0.0043 | 0.23 |
| PCS  PP (n=280)  CC (n=287)  ITT (n=368) |  |  |  |  |  |  |  |  |
|  | -0.076 | 0.13 | -0.047 | 0.32 | 0.11 | 0.13 | -0.0072 | 0.086 |
|  | -0.074 | 0.13 | -0.46 | 0.32 | 0.090 | 0.19 | -0.0061 | 0.14 |
|  | -0.064 | 0.14 | -0.041 | 0.33 | 0.079 | 0.19 | -0.0055 | 0.14 |
| MCS  PP (n=280)  CC (n=287  ITT (n=368) |  |  |  |  |  |  |  |  |
|  | 0.052 | 0.29 | 0.065 | 0.16 | 0.020 | 0.77 | 0.0088 | **0.034** |
|  | 0.052 | 0.29 | 0.060 | 0.19 | 0.024 | 0.72 | 0.0085 | **0.037** |
|  | 0.027 | 0.52 | 0.054 | 0.20 | 0.020 | 0.74 | 0.0047 | 0.20 |
| HADS^f^ |  |  |  |  |  |  |  |  |
| Depression  PP (n=280)  CC (n=287)  ITT (n=368) |  |  |  |  |  |  |  |  |
|  | 0.037 | 0.44 | 0.066 | 0.15 | -0.089 | 0.19 | 0.020 | **<0.0000** |
|  | 0.040 | 0.39 | 0.058 | 0.19 | -0.083 | 0.21 | 0.020 | **<0.0000** |
|  | 0.054 | 0.19 | 0.055 | 0.17 | -0.078 | 0.18 | 0.018 | **<0.0000** |
| Anxiety  PP (n=280)  CC (n=287)  ITT (n=368) |  |  |  |  |  |  |  |  |
|  | 0.032 | 0.51 | -0.0065 | 0.88 | -0.0032 | 0.63 | 0.022 | **<0.0000** |
|  | 0.030 | 0.53 | -0.014 | 0.75 | -0.017 | 0.79 | 0.022 | **<0.0000** |
|  | 0.031 | 0.44 | -0.021 | 0.60 | 0.00 | 1.0 | 0.020 | **0.012** |
| s-ED^g^  PP (n=280)  CC (n=287)  ITT (n=368) |  |  |  |  |  |  |  |  |
|  | -0.042 | 0.38 | -0.085 | 0.058 | 0.063 | 0.34 | -0.0053 | 0.18 |
|  | -0.028 | 0.56 | -0.079 | 0.078 | 0.044 | 0.50 | -0.0051 | 0.20 |
|  | -0.0090 | 0.82 | -0.80 | **0.043** | 0.040 | 0.47 | -0.0021 | 0.53 |
| PSS-14^h^  PP (n=280)  CC (n=287)  ITT(n=368) |  |  |  |  |  |  |  |  |
|  | -0.046 | 0.34 | -0.040 | 0.38 | -0.045 | 0.51 | -0.012 | **0.0031** |
|  | -0.043 | 0.37 | -0.036 | 0.42 | -0.037 | 0.58 | -0.013 | **0.0016** |
|  | -0.024 | 0.56 | -0.029 | 0.48 | -0.028 | 0.63 | -0.011 | **0.0033** |
| MADRS-S^i^  PP (n=280)  CC (n=287)  ITT (n=368) |  |  |  |  |  |  |  |  |
|  | 0.067 | 0.14 | 0.039 | 0.36 | -0.11 | 0.078 | -0.029 | **<0.000** |
|  | 0.066 | 0.15 | 0.040 | 0.34 | -0.010 | 0.10 | -0.029 | **<0.000** |
|  | 0.096 | **0.017** | 0.027 | 0.49 | -0.096 | 0.091 | -0.021 | **<0.000** |
| MRS^j^ |  |  |  |  |  |  |  |  |
| Somatic  PP (n=280)  CC (n=287)  ITT(n=368) |  |  |  |  |  |  |  |  |
|  | -0.11 | **0.028** | -0.040 | 0.38 | 0.054 | 0.43 | 0.00057 | 0.89 |
|  | -0.12 | **0.016** | -0.045 | 0.33 | 0.072 | 0.29 | 0.00026 | 0.29 |
|  | -0.096 | **0.020** | -0.052 | 0.21 | 0.061 | 0.30 | 0.0020 | 0.58 |
| Urogenital  PP (n=280)  CC (n=287)  ITT (n=368) |  |  |  |  |  |  |  |  |
|  | -0.12 | **0.015** | -0.13 | **0.0051** | 0.089 | 0.19 | -0.0031 | 0.43 |
|  | -0.11 | **0.018** | -0.13 | **0.0036** | 0.081 | 0.22 | -0.0019 | 0.61 |
|  | -0.094 | **0.020** | -0.13 | **0.0014** | 0.084 | 0.14 | -0.0012 | 0.73 |
| Psychological  PP (n=280)  CC (n=287)  ITT (n=368) |  |  |  |  |  |  |  |  |
|  | -0.11 | **0.024** | -0.10 | **0.029** | 0.035 | 0.60 | -0.0028 | 0.49 |
|  | -0.11 | **0.022** | -0.097 | **0.033** | 0.035 | 0.61 | -0.0033 | 0.40 |
|  | -0.072 | 0.080 | -0.11 | **0.0092** | 0.045 | 0.43 | -0.00097 | 0.79 |
| Total  PP (n=280)  CC (n=287)  ITT (n=368) |  |  |  |  |  |  |  |  |
|  | -0.13 | **0.0070** | -0.10 | **0.024** | 0.053 | 0.43 | -0.0022 | 0.58 |
|  | -0.13 | **0.0065** | -0.11 | **0.018** | 0.056 | 0.40 | -0.0018 | 0.66 |
|  | -0.11 | **0.011** | -012 | **0.004** | 0.065 | 0.27 | 0.00085 | 0.82 |
| ^a^Difference between 6-months follow-up and baseline using ranks with Bloms transformation as dependent variable.  ^b^ Short form health survey (SF-36). Physical component score (PCS) and mental component score (MCS). Positive values indicate increase reflect a better Health Related Quality of Life.  ^c^ Per Protocol. Received allocated intervention and responded to follow-up survey.  ^d^ Complete Case. Responded to follow-up survey  ^e^ Intention To Treat. All participants included with last outcome carried forward (LOCF).  ^f^ Hospital Anxiety and Depression scale (HADS). Negative values indicate decreased anxiety and depression.  ^g^ Self-rated Exhaustion Disorder (s-ED) identifies risk to develop exhaustion disorder with reduced workability and increased risk of sick leave. Negative values indicate decreased risk. ^h^ Perceived Stress Scale 14 (PSS-14). Negative values indicate decreased mental stress.  ^i^ Montgomery-Asberg Depression Rating Scale (MADRS-S) scoring. Negative values indicate decrease in depression. ^j^ Menopause Rating Scale (MRS) measure prevalence and severity of aging signs and Health Related Quality of Life- MRS subscale: Somatic symptoms - hot flushes, heart discomfort, sleeping problems and muscle and joint problems, Psychological symptoms - depressive mood, irritability, anxiety and physical and mental exhaustion, Urogenital symptoms - sexual problems, bladder problems and vaginal dryness, Total score - all subscales added. Negative values indicate improved Health Related Quality of Life. | | | | | | | | |

| **Table S3. The effect of group education and person-centered individual support at 6-month follow-up using ordinal regression.** | | | | | | | | |
| --- | --- | --- | --- | --- | --- | --- | --- | --- |
|  | | | | | | | | |
|  | Group education (GE) | | Person-centered Individual support (PCS) | | Interaction between GS and PCS | | Age (years) | |
|  | β (95% CI) | p-value | β (95% CI) | p-value | β (95% CI) | p-value | β (95% CI) | p-value |
| SF-36^a,b^ |  |  |  |  |  |  |  |  |
| Physical Function (PF)  PP^c^ (n=280) CC^d^ (n=287)  ITT^e^ (n=368) |  |  |  |  |  |  |  |  |
|  | -0.54 (-1.2 ↔ 0.089) | 0.093 | -0.18 (-0.77 ↔ 0.42) | 0.56 | 0.99 (0.11 ↔ 1.9) | **0.028** | 0.0035 (-0.050 ↔ 0.057) | 0.86 |
|  | -0.50 (-1.1 ↔ 0.12) | 0.11 | -0.19 (-0.76 ↔ 0.40) | 0.53 | 0.97 (0.10 ↔ 1.8) | **0.028** | 0.0046 (-0.048 ↔ 0.057) | 0.86 |
|  | -0.48 (-1.0 ↔ 0.076) | 0.091 | -0.14 (-0.68 ↔ 0.41) | 0.63 | 0.81 (0.020 ↔ 1.6) | **0.044** | 0.00077 (-0.049 ↔ 0.048) | 0.98 |
| Role Physical (RP)  PP (n=280)  CC (n=287)  ITT (n=368) |  |  |  |  |  |  |  |  |
|  | 0.070 (-0.57 ↔ 0.71) | 0.83 | 0.41 (-0.19 ↔ 1.0) | 0.18 | -0.093 (-0.98 ↔ 0.79) | 0.84 | -0.030 (-0.083 ↔ 0.024) | 0.28 |
|  | 0.062 (-0.57 ↔ 0.69) | 0.85 | 0.39 (-0.20 ↔ 0.98) | 0.19 | -0.094 (-0.97 ↔ 0.78) | 0.83 | -0.027 (-0.080 ↔ 0.026) | 0.32 |
|  | 0.024 (-0.54 ↔ 0.59) | 0.93 | 0.39 (-0.17 ↔ 0.96) | 0.17 | -0.14 (-0.94 ↔ 0.66) | 0.74 | -0.031 (-0.081 ↔ 0.019) | 0.23 |
| Bodily Pain (BP)  PP (n=280)  CC (n=287)  ITT (n=368) |  |  |  |  |  |  |  |  |
|  | -0.46 (-1.1 ↔ 1.2) | 0.15 | -0.33 (-0.93 ↔ 0.26) | 0.27 | 0.75 (-0.13 ↔ 1.6) | 0.094 | -0.029 (-0.082 ↔ 0.025) | 0.29 |
|  | -0.37 (-0.99 ↔ 0.26) | 0.25 | -0.31 (-0.90 ↔ 0.28) | 0.30 | 0.54 (-0.32 ↔ 1.4) | 0.22 | -0.018 (-0.071 ↔ 0.034) | 0.25 |
|  | -0.35 (-0.89 ↔ 0.19) | 0.21 | -0.24 (-0.77 ↔ 0.30) | 0.38 | 0.43 (-0.34 ↔ 1.2) | 0.27 | -0.022 (-0.70 ↔ 0.025) | 0.35 |
| General Health (GH)  PP (n=280)  CC (n=287)  ITT (n=368) |  |  |  |  |  |  |  |  |
|  | -0.28 (-0.93 ↔ 0.37) | 0.40 | 0.075 (-0.54 ↔ 0.69) | 0.81 | 0.48 (-0.44 ↔ 1.4) | 0.31 | 0.0016 (-0.057 ↔ 0.054) | 0.96 |
|  | -0.33 (-0.97 ↔ 0.32) | 0.32 | 0.081 (-0.53 ↔ 0.70) | 0.80 | 0.42 (-0.48 ↔ 1.3) | 0.36 | 0.0071 (-0.048 ↔ 0.062) | 0.80 |
|  | -0.30 (-0.84 ↔ 0.23) | 0.27 | 0.15 (-0.39 ↔ 0.68) | 0.59 | 0.25 (-0.51 ↔ 1.0) | 0.51 | 0.00040 (-0.048 ↔ 0.047) | 0.99 |
| Vitality (VT)  PP (n=280)  CC (n=287)  ITT (n=368) |  |  |  |  |  |  |  |  |
|  | -0.55 (-1.2 ↔ 0.12) | 0.11 | -0.10 (-0.75 ↔ 0.54) | 0.75 | 1.1 (0.093 ↔ 2.0) | **0.032** | -0.00097 (-0.059 ↔ 0.058) | 0.97 |
|  | -0.50 (-1.2 ↔ 0.16) | 0.14 | -0.12 (-0.76 ↔ 0.52) | 0.71 | 1.0 (0.046 ↔ 2.0) | **0.040** | -0.0063 (-0.064 ↔ 0.051) | 0.83 |
|  | - 0.48 (-1.0 ↔ 0.061) | 0.082 | -0.0036 (-0.55 ↔ 0.54) | 0.99 | 0.68 (-0.10 ↔ 1.4) | 0.088 | -0.014 (-0.062 ↔ 0.034) | 0.57 |
| Social function (SF)  PP (n=280)  CC (n=287)  ITT (n=368) |  |  |  |  |  |  |  |  |
|  | -0.12 (-0.75 ↔ 0.52) | 0.72 | 0.036 (-0.56 ↔ 0.63) | 0.91 | 0.42 (-0.47 ↔ 1.3) | 0.36 | 0.0027 (-0.051 ↔ 0.057) | 0.92 |
|  | -0.17 (-0.79 ↔ 0.46) | 0.60 | 0.12 (-0.58 ↔ 0.60) | 0.97 | 0.47 (-0.41 ↔ 1.4) | 0.29 | 0.0048 (-0.048 ↔ 0.058) | 0.86 |
|  | -0.20 (-0.74 ↔ 0.34) | 0.47 | 0.13 (-0.42 ↔ 0.67) | 0.66 | 0.28 (-0.49 ↔ 1.0) | 0.48 | 0.010 (-0.038 ↔ 0.058) | 0.68 |
| Role Emotional (RE)  PP (n=280)  CC (n=287)  ITT (n=368) |  |  |  |  |  |  |  |  |
|  | 0.18 (-0.47 ↔ 0.82) | 0.59 | 0.72 (-0.53 ↔ 0.67) | 0.82 | 0.70 (-0.20 ↔ 1.6) | 0.13 | 0.055 (6.2 ↔ 1.1) | **0.050** |
|  | 0.20 (-0.44 ↔ 0.83) | 0.55 | 0.60 (-0.54 ↔ 0.66) | 0.84 | 0.76 (-0.13 ↔ 1.6) | 0.095 | 0.058 (0.039 ↔ 0.11) | **0.035** |
|  | 0.25 (-0.030 ↔ 0.80) | 0.38 | 0.26 (-0.29 ↔ 0.80) | 0.36 | 0.26 (-0.52 ↔ 1.1) | 0.52 | 0.037 (-0.012 ↔ 0.085) | 0.14 |
| Mental Health (MH)  PP (n=280)  CC (n=287)  ITT (n=368) |  |  |  |  |  |  |  |  |
|  | 0.48 (-0.18 ↔ 1.1) | 0.15 | 0.69 (0.065 ↔ 1.3) | **0.030** | -0.18 (-1.1 ↔ 0.77) | 0.71 | 0.020 (-0.037 ↔ 0.078) | 0.48 |
|  | 0.48 (-0.17 ↔ 1.1) | 0.15 | 0.63 (0.13 ↔ 1.2) | **0.045** | -0.23 (-1.2 ↔ 0.70) | 0.63 | 0.035 (-0.031 ↔ 0.081) | 0.38 |
|  | 0.34 (-0.20 ↔ 0.87) | 0.21 | 0.65 (0.11 ↔ 1.2) | **0.018** | -0.30 (-1.07 ↔ 0.46) | 0.44 | 0.015 (-0.032 ↔ 0.063) | 0.53 |
| PCS  PP (n=280)  CC (n=287)  ITT (n=368) |  |  |  |  |  |  |  |  |
|  | -0.57 (-1.3 ↔ 0.11) | 0.10 | -0.41 (-1.0 ↔ 1.1) | 0.21 | 1.0 (0.095 ↔ 2.0) | **0.031** | -0.029 (-0.087 ↔ 0.029) | 0.33 |
|  | -0.59 (-1.3 ↔ 0.83) | 0.085 | -0.36 (-0.99 ↔ 0.28) | 0.27 | 0.94 (7.1 ↔ 1.9) | **0.050** | -0.023 (-0.079 ↔ 0.034) | 0.44 |
|  | -0.32 (-9.0 ↔ 0.26) | 0.28 | -0.17 (-0.75 ↔ 0.40) | 0.55 | 0.50 (-0.31 ↔ 1.3) | 0.23 | -0.025 (-0.075 ↔ 0.026) | 0.34 |
| MCS  PP (n=280)  CC(n=287)  ITT (n=368) |  |  |  |  |  |  |  |  |
|  | 0.19 (-0.51 ↔ 0.89) | 0.60 | 0.27 (-0.39 ↔ 0.93) | 0.42 | 0.29 (-0.72 ↔ 1.3) | 0.57 | 0.004 (0.056 ↔ 0.065) | 0.89 |
|  | 0.13 (-0.54 ↔ 0.79) | 0.71 | -0.090 (-0.73 ↔ 0.55) | 0.78 | -0.0014 (-0.93 ↔ 0.93) | 1.0 | 0.11 (0.056 ↔ 0.17) | 0.00011 |
|  | 0.15 (-0.45 ↔ 0.74) | 0.63 | 0.14 (-0.45 ↔ 0.73) | 0.65 | 0.76 (-0.78 ↔ 0.93) | 0.86 | 0.0054 (-0.048 ↔ 0.058) | 0.84 |
| HADS^f, g^ |  |  |  |  |  |  |  |  |
| Depression  PP (n=280)  CC (n=287)  ITT (n=368) |  |  |  |  |  |  |  |  |
|  | 0.50 (-0.18 ↔ 1.2) | 0.15 | 0.56 (-0.072 ↔ 1.2) | 0.082 | -0.80 (-1.7 ↔ 0.13) | 0.082 | 0.10 (0.046 ↔ 0.16) | **0.00044** |
|  | 0.50 (-0.17 ↔1.2) | 0.14 | 0.48 (-0.15 ↔ 1.1) | 0.14 | -0.76 (-1.7 ↔ 0.16) | 0.11 | 0.10 (0.044 ↔ 0.16) | **0.00050** |
|  | 0.47 (-0.076 ↔ 1.0) | 0.092 | 0.40 (-0.14 ↔ 0.94) | 0.15 | -0.59 (-1.4 ↔ 0.17) | 0.13 | 0.093 (0.045 ↔ 0.14) | **0.00023** |
| Anxiety  PP (n=280)  CC (n=287)  ITT (n=368) |  |  |  |  |  |  |  |  |
|  | 0.15 (-0.53 ↔ 0.83) | 0.67 | -0.040 (-0.68 ↔ 0.60) | 0.90 | -0.14 (-1.1 ↔ 0.81) | 0.78 | 0.12 (0.061 ↔ 0.18) | **0.000067** |
|  | -0.078 (-0.74 ↔ 0.58) | 0.81 | -0.018 (-0.64 ↔ 0.60) | 0.95 | -0.23 (-1.1 ↔ 0.70) | 0.63 | -0.068 (-0.12 ↔ -0.011) | **0.019** |
|  | 0.16 (-0.38 ↔ 0.71) | 0.55 | -0.15 (-0.69 ↔ 0.40) | 0.59 | 0.086 (-0.68 ↔ 0.85) | 0.83 | 0.11 (0.059 ↔ 0.16) | **0.000015** |
| s-ED^f,h^  PP (n=280)  CC (n=287)  ITT (n=368) |  |  |  |  |  |  |  |  |
|  | -0.16 (-0.81 ↔ 0.49) | 0.64 | -0.52 (-1.1 ↔ 0.94) | 0.097 | 0.29 (-0.63 ↔ 1.2) | 0.54 | -0.028 (-0.084 ↔ 0.027) | 0.32 |
|  | -0.053 (-0.69 ↔ 0.59) | 0.87 | -0.47 (-1.1 ↔ 0.14) | 0.13 | 0.16 (-0.74 ↔ 1.6) | 0.73 | -0.027 (-0.082 ↔ 0.027) | 0.33 |
|  | -0.096 (-0.73 ↔ 0.53) | 0.76 | -0.65 (-1.3 ↔ -0.020) | **0.043** | 0.29 (-0.59 ↔ 1.1) | 0.52 | -0.23 (-0.78 ↔ 0.032) | 0.41 |
| PSS-14^f,i^  PP (n=280)  CC (n=287)  ITT (n=368) |  |  |  |  |  |  |  |  |
|  | -0.068 (-0.74 ↔ 0.60) | 0.84 | -0.028 (-0.61 ↔ 0.60) | 0.93 | -0.31 (-1.3 ↔ 0.62) | 0.51 | -0.059 (-0.12 ↔ -0.0014) | **0.045** |
|  | -0.078 (-0.74 ↔ 0.58) | 0.82 | -0.018 (-0.64 ↔ 0.60) | 0.95 | -0.23 (-1.1 ↔ 0.70) | 0.63 | -0.068 (-0.12 ↔ -0.011) | **0.019** |
|  | -0.034 (-0.57 ↔ 0.50) | 0.90 | -0.057 (-0.59 ↔ 0.48) | 0.83 | -0.16 (-0.92 ↔ 0.60) | 0.67 | -0.057 (-0.11 ↔ -0.010) | **0.018** |
| MADRS^f,j^  PP (n=280)  CC (n=287)  ITT (n=368) |  |  |  |  |  |  |  |  |
|  | 1.6 (0.75 ↔ 2.5) | **0.00029** | 1.1 (0.28 ↔ 2.0) | **0.0091** | -2.1 (-3.3 ↔ -0.92) | **0.00054** | -0.20 (-0.28 ↔ -0.12) | **<0.0000** |
|  | 1.6 (0.69 ↔ 2.5) | **0.000073** | 1.1 (0.24 ↔ 1.6) | **0.012** | -1.8 (-3.0 ↔ -0.67) | **0.0021** | -0.20 (-0.28 ↔ -0.13) | **<0.0000** |
|  | 1.1 (0.46 ↔ 1.6) | **0.00049** | 0.47 (-0.13 ↔ 1.1) | 0.13 | -1.0 (-1.8 ↔ -0.17) | **0.018** | -0.11 (-0.1 6 ↔ -0.058) | **0.00040** |
| MRS^f,k^ |  |  |  |  |  |  |  |  |
| Somatic  PP (n=280)  CC (n=287)  ITT (n=368) |  |  |  |  |  |  |  |  |
|  | -0.84 (-1.5 ↔ -0.18) | **0.013** | -0.47 (-1.1 ↔ 0.13) | 0.12 | 0.42 (-0.50 ↔ 1.3) | 0.37 | 0.026 (-0.030 ↔ 0.081) | 0.37 |
|  | -0.91 (-1.6 ↔ -0.26) | **0.0059** | -0.49 (-1.1 ↔ 0.11) | 0.11 | 0.56 (-0.35 ↔ 1.5) | 0.23 | 0.026 (-0.029 ↔ 0.080 | 0.35 |
|  | -0.69 (-1.2 ↔ -0.15) | **0.012** | -0.47 (-1.0 ↔ 0.64) | 0.084 | 0.44 (-0.32 ↔ 1.2) | 0.26 | 0.030 (-0.017↔ 0.078 | 0.21 |
| Urogenital  PP (n=280)  CC (n=287)  ITT (n=368) |  |  |  |  |  |  |  |  |
|  | -0.85 (-1.5 ↔ -0.22) | **0.0087** | -0.92 (-1.5 ↔ -0.32) | **0.0025** | 0.71 (-0.18 ↔ 1.6) | 0.12 | -0.014(-0.067 ↔ 0.040) | 0.61 |
|  | -0.83 (-1.5 ↔ -0.20) | **0.010** | -0.93 (-1.5 ↔ -0.34) | **0.0020** | 0.64 (-0.24 ↔ -1.5 | 0.15 | -0.0066 (-0.059 ↔ -0.046) | 0.86 |
|  | -0.69 (-1.2 ↔ -0.14) | **0.013** | -0.94 (-1.5 ↔ -0.39 | **0.0077** | 0.67 (-0.10 ↔ 1.4 | 0.089 | -0.0041 (-0.052 ↔ 0.044) | 0.86 |
| Psychological  PP (n=280)  CC (n=287)  ITT (n=368) |  |  |  |  |  |  |  |  |
|  | -0.63 (-1.3 ↔ 0.023) | 0.058 | -0.84 (-1.5 ↔ -0.21) | **0.0087** | 0.37 (-0.58 ↔ 1.3) | 0.45 | 0.00044 (-0.058 ↔ 0.057) | 0.99 |
|  | -0.63 (-1. 3 ↔ 0.013) | 0.055 | -0.83 (-1.4 ↔ -0.21) | **0.0087** | 0.39 (-0.55 ↔ 1.3) | 0.42 | -0.0084 (-0.065 ↔ 0.048) | 0.77 |
|  | -0.41 (-0.95 ↔ -0.13) | 0.13 | -0.85 (-1.4 ↔ -0.30) | **0.0024** | 0.47 (0.31 ↔ -1.2) | 0.24 | 0.0045 (-0.044 ↔ -0.053) | 0.85 |
| Total  PP (n=280)  CC (n=287)  ITT (n=368) |  |  |  |  |  |  |  |  |
|  | -1.1 (-1.8 ↔ -0.40) | **0.0019** | -0.91 (-1.6 ↔ -0.28) | **0.0048** | 0.36 (-0.66 ↔ 1.4) | 0.49 | 0.027 (-0.034 ↔ 0.088) | 0.38 |
|  | -1.1 (-1.8 ↔ -0.40) | **0.0019** | -0.95 (-1.6 ↔ -0.31) | **0.0033** | 0.35 (-0.66 ↔ 1.4) | 0.50 | 0.025 (-0.034 ↔ -0.085) | 0.41 |
|  | -0.78 (-1.3 ↔ -0.23) | **0.0051** | -0.91 (-1.5 ↔ -0.37) | **0.0010** | 0.51 (-0.28 ↔ 1.3) | 0.20 | 0.028 (-0.021 ↔ 0.077) | 0.26 |
| ^a^ Dependent variables transformed where coded as improvement +1, worsening as -1 and unchanged as 0.  ^b^ Short form health survey (SF-36). Physical component score (PCS) and mental component score (MCS). Positive values indicate increase reflect a better Health Related Quality of Life.  ^c^ Per Protocol. Received allocated intervention and responded to follow-up survey.  ^d^ Complete Case. Responded to follow-up survey.  ^e^ Intention To Treat. All participants included with last outcome carried forward (LOCF).  ^f^ Dependent variables transformed where improvement is coded as -1 worsening as +1 and unchanged as 0.  ^g^ Hospital Anxiety and Depression Scale (HADS). Negative values indicate decreased anxiety and depression.  ^h^ Self-rated Exhaustion Disorder (s-ED) identifies risk to develop exhaustion disorder with reduced workability and increased risk of sick leave.  Negative values indicate decreased risk.  ^i^ Perceived Stress Scale 14 (PSS-14). Negative values indicate decreased mental stress.  ^j^ Montgomery-Asberg Depression Rating Scale (MADRS-S) scoring. Negative values indicate decrease in depression.  ^k^ Menopause Rating Scale (MRS) measure prevalence and severity of aging signs and Health Related Quality of Life. MRS subscale: Somatic symptoms - hot flushes, heart discomfort, sleeping problems and muscle and joint problems, Psychological symptoms - depressive mood, irritability, anxiety and physical and mental exhaustion, Urogenital symptoms - sexual problems, bladder problems and vaginal dryness, Total score - all subscales added. Negative values indicate improved Health Related Quality of Life. | | | | | | | | |

| **Table S4. The effect of group education and person-centered individual support at 12-month follow-up using linear regression.** | | | | | | | | |
| --- | --- | --- | --- | --- | --- | --- | --- | --- |
|  | Group education  (GE) | | Person-centered Individual support (PCS) | | Interaction between GS and PCS | | Age (years) | |
|  | β^a^ | p-value | β^a^ | p-value | β^a^ | p-value | β^a^ | p-value |
| SF-36^b^ |  |  |  |  |  |  |  |  |
| Physical Function (PF)  PP^c^ (n=284) CC^d^ (n=289)  ITT^e^ (n=368) |  |  |  |  |  |  |  |  |
|  | -0.041 | 0.38 | 0.024 | 0.60 | 0.038 | 0.58 | 0.0026 | 0.51 |
|  | -0.026 | 0.59 | 0.029 | 0.53 | 0.017 | 0.80 | 0.0022 | 0.59 |
|  | -0.019 | 0.64 | 0.044 | 0.27 | -0.00068 | 0.99 | 0.0015 | 0.66 |
| Role Physical (RP)  PP (n=284)  CC n=289)  ITT (n=368) |  |  |  |  |  |  |  |  |
|  | -0.017 | 0.72 | 0.063 | 0.15 | -0.034 | 0.61 | -0.0013 | 0.73 |
|  | -0.017 | 0.72 | 0.059 | 0.18 | -0.036 | 0.58 | -0.00096 | 0.81 |
|  | 0.0029 | 0.94 | 0.066 | 0.088 | -0.068 | 0.22 | -0.0023 | 0.49 |
| Bodily Pain (BP)  PP (n=284)  CC (n=289)  ITT (n=368) |  |  |  |  |  |  |  |  |
|  | -0.11 | **0.021** | 0.047 | 0.30 | 0.066 | 0.33 | 2.4 | 1.0 |
|  | -0.098 | **0.043** | 0.061 | 0.18 | 0.049 | 0.46 | 0.00014 | 0.97 |
|  | -0.078 | 0.059 | 0.071 | 0.083 | 0.0031 | 0.96 | 0.0094 | 0.79 |
| General Health (GH)  PP (n=284)  CC (n=289)  ITT (n=368) |  |  |  |  |  |  |  |  |
|  | -0.099 | **0.046** | 0.025 | 0.58 | 0.084 | 0.22 | 0.00016 | 0.97 |
|  | -0.084 | 0.085 | 0.035 | 0.45 | 0.065 | 0.34 | 0.00017 | 0.97 |
|  | -0.076 | 0.069 | 0.051 | 0.22 | 0.034 | 0.56 | 0.00019 | 0.96 |
| Vitality (VT)  PP (n=284)  CC (n=289)  ITT (n=368) |  |  |  |  |  |  |  |  |
|  | 0.019 | 0.70 | 0.086 | 0.062 | 0.019 | 0.78 | -0.00082 | 0.84 |
|  | 0.0045 | 0.93 | 0.088 | 0.057 | 0.024 | 0.72 | 0.00068 | 0.87 |
|  | 0.0031 | 0.94 | 0.098 | **0.019** | -0.021 | 0.72 | -0.0095 | 0.80 |
| Social function (SF)  PP (n=284)  CC (n=289)  ITT (n=368) |  |  |  |  |  |  |  |  |
|  | 0.089 | 0.066 | 0.10 | **0.025** | -0.066 | 0.33 | 0.0018 | 0.65 |
|  | 0.068 | 0.16 | 0.099 | **0.029** | -0.044 | 0.51 | 0.0019 | 0.64 |
|  | 0.036 | 0.39 | 0.077 | 0.062 | -0.040 | 0.50 | 0.0030 | 0.41 |
| Role Emotional RE  PP (n=284)  CC (n=289)  ITT (n=368) |  |  |  |  |  |  |  |  |
|  | 0.026 | 0.57 | 0.053 | 0.22 | 0.024 | 0.70 | 0.0092 | **0.018** |
|  | 0.050 | 0.29 | 0.049 | 0.27 | -0.051 | 0.44 | 0.0086 | **0.034** |
|  | 0.032 | 0.41 | 0.060 | 0.13 | 0.0030 | 0.96 | 0.0058 | 0.093 |
| Mental Health (MH)  PP (n=284)  CC (n=289)  ITT (n=368) |  |  |  |  |  |  |  |  |
|  | 0.051 | 0.30 | 0.13 | **0.0063** | -0.035 | 0.60 | 0.0030 | 0.46 |
|  | 0.024 | 0.62 | 0.13 | **0.0044** | -0.011 | 0.86 | 0.0045 | 0.27 |
|  | 0.010 | 0.81 | 0.14 | **0.0012** | -0.051 | 0.39 | 0.0016 | 0.66 |
| PCS  PP (n=284)  CC (n=289)  ITT (n=368) |  |  |  |  |  |  |  |  |
|  | 0.10 | **0.041** | 0.0078 | 0.87 | 0.052 | 0.45 | -0.0021 | 0.61 |
|  | -0.086 | 0.083 | 0.011 | 0.82 | 0.045 | 0.51 | -0.0037 | 0.37 |
|  | -0.059 | 0.17 | 0.045 | 0.29 | -0.027 | 0.65 | -0.020 | 0.58 |
| MCS  PP (n=284)  CC (n=289)  ITT (n=368) |  |  |  |  |  |  |  |  |
|  | 0.071 | 0.14 | 0.12 | **0.012** | -0.042 | 0.54 | 0.0079 | 0.057 |
|  | 0.051 | 0.29 | 0.12 | **0.011** | -0.025 | 0.71 | 0.0093 | **0.023** |
|  | 0.036 | 0.40 | 0.12 | **0.0064** | -0.042 | 0.48 | 0.0054 | 0.15 |
| HADS^f^ |  |  |  |  |  |  |  |  |
| Depression  PP (n=284)  CC (n=289)  ITT (n=368) |  |  |  |  |  |  |  |  |
|  | 0.037 | 0.44 | 0.032 | 0.46 | -0.070 | 0.28 | 0.024 | **<0.0000** |
|  | 0.037 | 0.42 | 0.030 | 0.49 | -0.068 | 0.29 | 0.025 | **<0.0000** |
|  | 0.032 | 0.44 | 0.015 | 0.72 | -0.046 | 0.43 | 0.021 | **<0.0000** |
| Anxiety  PP (n=284)  CC (n=289)  ITT (n=368) |  |  |  |  |  |  |  |  |
|  | 0.031 | 0.50 | -0.058 | 0.18 | -0.042 | 0.52 | 0.023 | **<0.0000** |
|  | 0.027 | 0.56 | -0.063 | 0.14 | -0.035 | 0.58 | 0.025 | **<0.0000** |
|  | 0.028 | 0.49 | -0.061 | 0.13 | -0.24 | 0.68 | 0.019 | **<0.0000** |
| s-ED^g^  PP (n=284)  CC (n=289)  ITT (n=368) |  |  |  |  |  |  |  |  |
|  | -0.021 | 0.67 | -0.051 | 0.28 | 0.038 | 0.59 | 0.0030 | 0.48 |
|  | -0.012 | 0.81 | -0.045 | 0.33 | 0.027 | 0.70 | 0.0019 | 0.64 |
|  | -0.0011 | 0.97 | -0.054 | 0.19 | 0.029 | 0.63 | 0.0044 | 0.23 |
| PSS-14^h^  PP (n=284)  CC (n=289)  ITT (n=368) |  |  |  |  |  |  |  |  |
|  | -0.050 | 0.30 | -0.15 | **0.0012** | 0.015 | 0.82 | -0.0019 | 0.63 |
|  | -0.045 | 0.35 | -0.15 | **0.00072** | 0.017 | 0.79 | -0.0031 | 0.44 |
|  | -0.049 | 0.24 | -0.14 | **0.00053** | 0.049 | 0.40 | -0.0030 | 0.39 |
| MADRS-S^i^  PP (n=284)  CC (n=289)  ITT (n=368) |  |  |  |  |  |  |  |  |
|  | -0.024 | 0.62 | -0.096 | **0.037** | -0.019 | 0.78 | -0.0046 | 0.26 |
|  | -0.017 | 0.72 | -0.10 | **0.024** | -0.023 | 0.74 | -0.004 | 0.31 |
|  | -0.004 | 0.91 | -0.097 | **0.019** | -0.012 | 0.84 | -0.0031 | 0.39 |
| MRS^j^ |  |  |  |  |  |  |  |  |
| Somatic  PP (n=284)  CC (n=289)  ITT (n=368) |  |  |  |  |  |  |  |  |
|  | -0.084 | 0.086 | -0.084 | 0.066 | 0.068 | 0.32 | -0.0023 | 0.57 |
|  | -0.057 | 0.25 | -0.081 | 0.081 | 0.050 | 0.46 | -0.0033 | 0.42 |
|  | -0.051 | 0.22 | -0.080 | 0.056 | 0.069 | 0.25 | -0.0020 | 0.57 |
| Urogenital  PP (n=284)  CC (n=289)  ITT (368) |  |  |  |  |  |  |  |  |
|  | -0.069 | 0.16 | -0.12 | **0.012** | 0.077 | 0.25 | 0.0031 | 0.44 |
|  | -0.044 | 0.36 | -0.11 | **0.015** | 0.058 | 0.38 | 0.0027 | 0.50 |
|  | -0.022 | 0.59 | -0.13 | **0.0022** | 0.043 | 0.47 | 0.0042 | 0.24 |
| Psychological  PP (n=284)  CC (n=289)  ITT (n=368) |  |  |  |  |  |  |  |  |
|  | -0.058 | 0.23 | -0.14 | **0.0016** | 0.038 | 0.57 | -0.0052 | 0.20 |
|  | -0.039 | 0.41 | -0.14 | **0.0017** | 0.026 | 0.70 | -0.0064 | 0.11 |
|  | -0.046 | 0.26 | -0.15 | **0.00021** | 0.061 | 0.29 | -0.0041 | 0.25 |
| Total  PP (n=284)  CC (n=289)  ITT (n=368) |  |  |  |  |  |  |  |  |
|  | -0.079 | 0.10 | -0.15 | **0.00078** | 0.077 | 0.25 | -0.0033 | 0.41 |
|  | -0.049 | 0.31 | -0.15 | **0.00097** | 0.059 | 0.38 | -0.0045 | 0.27 |
|  | -0.060 | 0.15 | -0.17 | **0.000076** | 0.097 | 0.098 | -0.0025 | 0.48 |
| ^a^ Difference between 12-months follow-up and baseline using ranks with Bloms transformation as dependent variable.  ^b^ Short form health survey (SF-36). Physical component score (PCS) and mental component score (MCS). Positive values indicate increase reflect a better Health Related Quality of Life.  ^c^ Per Protocol. Received allocated intervention and responded to follow-up survey.  ^d^ Complete Case. Responded to follow-up survey.  ^e^ Intention To Treat. All participants included with last outcome carried forward (LOCF).  ^f^ Hospital Anxiety and Depression scale (HADS). Negative values indicate decreased anxiety and depression.  ^g^ Self-rated Exhaustion Disorder (s-ED) identifies risk to develop exhaustion disorder with reduced workability and increased risk of sick leave.  Negative values indicate decreased risk.  ^h^ Perceived Stress Scale 14 (PSS-14). Negative values indicate decreased mental stress.  ^i^ Montgomery-Asberg Depression Rating Scale (MADRS-S) scoring. Negative values indicate decrease in depression.  ^j^ Menopause Rating Scale (MRS) measure prevalence and severity of aging-symptoms and Health Related Quality of Life. MRS subscale: Somatic symptoms - hot flushes, heart discomfort, sleeping problems and muscle and joint problems, Psychological symptoms - depressive mood, irritability, anxiety and physical and mental exhaustion, Urogenital symptoms - sexual problems, bladder problems and vaginal dryness, Total score - all subscales added. Negative values indicate improved Health-Related Quality of Life. | | | | | | | | |

| **Table S5. The effect of group education and person-centered individual support at 12-month follow-up using ordinal regression.** | | | | | | | | |
| --- | --- | --- | --- | --- | --- | --- | --- | --- |
|  | Group education  (GE) | | Person-centered Individual support (PCS) | | Interaction between GS and PCS | | Age (years) | |
|  | β (95% CI) | p-value | β (CI) | p-value | β (CI) | p-value | β (CI) | p-value |
| SF-36^a,b^ |  |  |  |  |  |  |  |  |
| Physical Function (PF)  PP^c^ (n=284) CC^d^ (n=289)  ITT^e^ (n=368) |  |  |  |  |  |  |  |  |
|  | -0.30 (-0.93 ↔ 0.32) | 0.34 | 0.099 (-0.48 ↔ 0.68) | 0.74 | 0.31 (-0.56 ↔1.2) | 0.48 | 0.013 (-0.039 ↔ 0.066) | 0.62 |
|  | -0.21 (-0.82 ↔ 0.41) | 0.51 | 0.13 (-0.46 ↔ 0.71 | 0.67 | 0.17 (-0.69 ↔1.0) | 0.69 | 0.010 (-0.043 ↔ 0.062) | 0.72 |
|  | -0.15 (-0.69 ↔ 0.39) | 0.60 | 0.24 (0.30 ↔ 0.77) | 0.39 | 0.047 (-0.71 ↔0.81) | 0.90 | 0.0086 (-0.039 ↔ 0.056) | 0.72 |
| Role Physical (RP)  PP (n=284)  CC (n=289)  ITT (n=368) |  |  |  |  |  |  |  |  |
|  | -0.088 (-0.72 ↔ 0.55) | 0.79 | 0.46 (-0.13 ↔ 1.1) | 0.13 | -0.34 (-1.2 ↔0.54) | 0.45 | -0.016 (-0.070 ↔ 0.037) | 0.55 |
|  | -0.092 (-0.72 ↔ 0.54) | 0.77 | 0.44 (-0.16 ↔ 1.0) | 0.15 | -0.35 (-1.2 ↔0.53) | 0.43 | -0.014 (-0.067 ↔ 0.040) | 0.62 |
|  | 0.034 (-0.53 ↔ 0.60) | 0.91 | 0.51 (-0.047 ↔ 1.1) | 0.073 | -0.55 (-1.3 ↔0.24) | 0.17 | -0.024 (-0.073 ↔ 0.025) | 0.34 |
| Bodily Pain (BP)  PP (n=284)  CC (n=289)  ITT(n=368) |  |  |  |  |  |  |  |  |
|  | -0.70 (-1.3 ↔ 0.061) | **0.032** | 0.37 (-0.24 ↔ 0.97) | 0.24 | 0.20 (-0.68 ↔ 1.1) | 0.65 | 0.0034 (-0.053 ↔ 0.054) | 0.99 |
|  | -0.65 (-1.3 ↔ -0.027) | **0.041** | 0.40 (-0.20 ↔ 1.0) | 0.19 | 0.15 (-0.72 ↔ 1.0) | 0.74 | 0.0018 (-0.023 ↔ 0.055) | 0.95 |
|  | -0.52 (-1.056 ↔ 0.15) | 0.057 | 0.48 (-0.064 ↔ 1.0) | 0.084 | -0.082 (-0.84 ↔ 0.68) | 0.83 | 0.0043 (-0.043 ↔ 0.052) | 0.86 |
| General Health (GH)  PP (n=284)  CC (n=289)  ITT(n=368) |  |  |  |  |  |  |  |  |
|  | -0.67 (-1.3 ↔ 0.022) | **0.043** | 0.22 (-0.40 ↔ 0.84) | 0.49 | 0.63 (-0.28 ↔ 1.5) | 0.18 | 0.0043 (-0.052 ↔ 0.060) | 0.88 |
|  | -0.54 (-1.2 ↔ 0.099) | 0.097 | 0.28 (-0.34 ↔ 0.90) | 0.38 | 0.46 (-0.44 ↔ 1.4) | 0.32 | 0.0050 (-0.051 ↔ 0.061) | 0.86 |
|  | -0.46 (-1.0 ↔ 0.076) | 0.092 | 0.38 (0.17 ↔ 0.92 | 0.17 | 0.20 (-0.57 ↔ 0.97) | 0.61 | 0.0040 (-0.044 ↔ 0.052) | 0.87 |
| Vitality (VT)  PP (n=284)  CC (n=289)  ITT (n=368) |  |  |  |  |  |  |  |  |
|  | 0.15 (-0.51 ↔ 0.81) | 0.66 | 0.48 (-0.15 ↔ 1.0) | 0.13 | -0.068 (-1.1 ↔ 0.87) | 0.88 | 0.017 (-0.040 ↔ 0.075) | 0.55 |
|  | 0.084 (-0.57 ↔ 0.73) | 0.80 | 0.52 (-0.11 ↔ 1.2) | 0.11 | -0.10 (-1.1 ↔ 0.83) | 0.83 | 0.024 (-0.034 ↔ 0.081) | 0.42 |
|  | 0.019 (-0.52 ↔ 0.56) | 0.95 | 0.62 (0.059 ↔ 1.2) | **0.030** | -0.35 (-1.1 ↔ 0.43) | 0.38 | 0.0040 (-0.044 ↔ 0.052) | 0.687 |
| Social function (SF)  PP (n=284)  CC (n=289)  ITT(n=368) |  |  |  |  |  |  |  |  |
|  | 0.47 (-0.16 ↔ 1.1) | 0.15 | 0.54 (-0.053 ↔ 1.1) | 0.074 | -0.38 (-1.3 ↔ 0.51) | 0.41 | 0.0033 (-0.051 ↔ 0.057) | 0.91 |
|  | 0.31 (-0.32 ↔ 0.93) | 0.34 | 0.51 (-0.083 ↔ 1.1) | 0.092 | -0.19 (-1.1 ↔ 0.69) | 0.68 | 0.0029 (-0.051 ↔ 0.057) | 0.92 |
|  | 0.16 (-0.39 ↔ 0.70) | 0.57 | 0.38 (-0.16 ↔ 0.92) | 0.17 | -0.24 (-1.0 ↔ 0.53) | 0.55 | 0.022 (-0.026 ↔ 0.072) | 0.37 |
| Role Emotional (RE)  PP (n=284)  CC (n=289)  ITT (n=368) |  |  |  |  |  |  |  |  |
|  | 0.20 (-0.45 ↔ 0.85) | 0.55 | 0.38 (-0.23 ↔ 0.98) | 0.22 | 0.24 (-0.66 ↔ 1.1) | 0.60 | 0.059 (-0.0040 ↔ 0.12) | **0.036** |
|  | -0.38 (-1.9 ↔ -1.1) | 0.61 | 0.81 (-0.30 ↔ 1.9) | 0.15 | -0.89 (-2.9 ↔ 1.1) | 0.38 | 0.034 (-0.75 ↔ 0.14) | 0.54 |
|  | 0.33 (-0.23 ↔ 0.88) | 0.25 | 0.45 (-0.10 ↔ 1.0) | 0.11 | 0.029 (-0.76 ↔ 0.81) | 0.94 | 0.034 (-0.015 ↔ 0.083) | 0.18 |
| **Mental Health (MH)**  **PP (n=284)**  **CC (n=289)**  **ITT (n=368)** |  |  |  |  |  |  |  |  |
|  | -0.015 (-0.65 ↔ 0.63) | 0.96 | 0.73 (0.094 ↔ 1.4) | **0.024** | -0.011 (-0.95 ↔ 0.92) | 0.98 | 0.0037 (-0.053 ↔ 0.060) | 0.90 |
|  | -0.17 (-0.80 ↔ 0.463) | 0.60 | 0.75 (0.12 ↔ 1.4) | **0.021** | 0.16 (-0.77 ↔ 1.1) | 0.73 | 0.010 (-0.047 ↔ 0.067) | 0.73 |
|  | -0.12 (-0.66 ↔ 0.41) | 0.65 | 0.81 (0.26 ↔ 1.4) | **0.0042** | -0.26 (-1.0 ↔ 0.52) | 0.51 | -0.0036 (-0.052 ↔ 0.045) | 0.88 |
| PCS  PP (n=280)  CC (n=289)  ITT (n=368) |  |  |  |  |  |  |  |  |
|  | -0.51 (-1.2 ↔ 0.17) | 0.14 | 0.21 (-0.43 ↔ 0.86) | 0.52 | 0.0077 (-0.94 ↔ 0.96) | 0.99 | 0.0040 (-0.054 ↔ 0.062) | 0.89 |
|  | -0.47 (-1.1 ↔ 0.20) | 0.17 | 0.21 (-0.43 ↔ 0.86) | 0.52 | 0.00029 (-0.94 ↔ 0.94) | 1.0 | -0.00078 (-0.066 ↔ 0.050) | 0.89 |
|  | -0.24 (-0.81 ↔ 0.34) | 0.42 | 0.54 (0.045 ↔ 1.1) | **0.0071** | -0.56 (-1.4 ↔ 0.26) | 0.18 | -0.012 (-0.063 ↔ 0.039) | 0.65 |
| MCS  PP (n=280)  CC (n=289)  ITT (n=368) |  |  |  |  |  |  |  |  |
|  | 0.22 (-0.46 ↔ 0.91) | 0.51 | 0.97 (0.28 ↔ 1.6) | **0.0053** | -0.31 (-1.3 ↔ 0.69) | 0.54 | 0.021 (-0.040 ↔ 0.082) | 0.51 |
|  | 0.18 (-0.40 ↔ 0.85) | 0.61 | 1.0 (0.35 ↔ 1.7) | **0.0031** | -0.31 (-1.3 ↔ 0.69) | 0.54 | 0.027 (-0.034 ↔ 0.088) | 0.39 |
|  | 0.37 (-0.27 ↔ 0.90) | 0.29 | 0.84 (0.24 ↔ 1.5) | **0.0067** | -0.51 (-1.3 ↔ 0.36) | 0.25 | 0.0083 (-0.045 ↔ 0.062) | 0.76 |
| HADS^f,g^ |  |  |  |  |  |  |  |  |
| Depression  PP (n=284)  CC (n=289)  ITT (n=368) |  |  |  |  |  |  |  |  |
|  | 0.17 (-0.52 ↔ 0.86) | 0.63 | 0.18 (-0.47 ↔ 0.82) | 0.59 | -0.29 (-1.2 ↔ 0.66) | 0.56 | 0.15 (0.084 ↔ 0.21) | **<0.0000** |
|  | 0.14 (-0.54 ↔ 0.83) | 0.68 | 0.16 (-0.48 ↔ 0.81) | 0.61 | -0.27 (-1.2 ↔ 0.68) | 0.58 | 0.16 (0.094 ↔ 0.22) | **<0.0000** |
|  | 0.10 (-0.45 ↔ 0.65) | 0.71 | -0.48 (0.60 ↔ 0.50) | 0.86 | -0.089 (-0.86 ↔ 0.69) | 0.82 | 0.13 (0.075 ↔ 0.18) | **<0.0000** |
| Anxiety  PP (n=284)  CC (n=287)  ITT (n=368) |  |  |  |  |  |  |  |  |
|  | 0.16 (-0.51 ↔ 0.84) | 0.64 | -0.44 (-1.1 ↔ 0.21) | 0.19 | -0.49 (-1.4 ↔ 0.49) | 0.33 | 0.11 (0.048 ↔ 0.17) | **<0.00044** |
|  | 0.13 (-0.54 ↔ -0.80) | 0.70 | -0.46 (-1.1 ↔ 0.19) | 0.17 | -0.44 (-1.4 ↔ 0.53) | 0.37 | 0.12 (0.056 ↔ 0.18) | **<0.00017** |
|  | 0.085 (-0.45 ↔ 0.63) | 0.76 | -0.48 (-1.0 ↔ 0.071) | 0.088 | -0.10 (-0.88 ↔ 0.89) | 0.80 | -0.089 (0.039 ↔ 0.14) | **<0.00044** |
| s-ED^f,h^  PP (n=284)  CC (n=289)  ITT (n=368) |  |  |  |  |  |  |  |  |
|  | -0.61 (-1.3 ↔ 0.12) | 0.10 | -0.49 (-1.2 ↔ 0.18) | 0.15 | 0.74 (-0.27 ↔ 1.8) | 0.15 | 0.0082 (-0.053 ↔ 0.069) | 0.79 |
|  | -0.54 (-1.3 ↔ 0.18) | 0.15 | -0.48 (-1.1 ↔ 0.19) | 0.16 | 0.72 (-0.29 ↔ 1.7) | 0.16 | 0.00045 (-0.061 ↔ 0.062) | 0.99 |
|  | -0.24 (-0.80 ↔ 0.32) | 0.40 | -0.44 (-1.0 ↔ 0.13) | 0.13 | 0.39 (-0.41 ↔ 1.2) | 0.34 | 0.035 (-0.015 ↔ 0.085) | 0.17 |
| PSS-14^f,i^  PP (n=284)  CC (n=289)  ITT (n=368) |  |  |  |  |  |  |  |  |
|  | -0.32 (-0.98 ↔ 0.35) | 0.35 | -0.93 (-1.6 ↔ 0.29) | **0.0045** | -0.022 (-1.0 ↔ 0.95) | 0.97 | -0.0087 (-0.068 ↔ 0.050) | 0.77 |
|  | -0.33 (-0.98 ↔ 0.33) | 0.33 | -0.99 (-1.6 ↔ 0.34) | **0.0026** | 0.049 (-0.92 ↔ -1.01) | 0.92 | -0.014 (-0.073 ↔ 0.045) | 0.63 |
|  | -0.31 (-0.85 ↔ 0.23) | 0.26 | -0.92 (-1.5 ↔ -0.37) | **0.0011** | 0.34 (-0.44 ↔ 1.1) | 0.39 | -0.017 (-0.065 ↔ 0.032) | 0.50 |
| MADRS-S^f,j^  PP (n=284)  CC (n=289)  ITT (n=368) |  |  |  |  |  |  |  |  |
|  | 0.059 (-0.58 ↔ 0.70) | 0.86 | -0.53 (-1.1 ↔ 0.71) | 0.083 | -0.18 (-1.1 ↔ 0.73) | 0.70 | -0.025 (-0.080 ↔ 0.030) | 0.38 |
|  | 0.16 (-0.48 ↔ 0.79) | 0.63 | -0.56 (-1.2 ↔ 0.048) | 0.071 | -0.26 (-1.2 ↔ 0.64) | 0.57 | -0.020 (-0.075 ↔ 0.036) | 0.49 |
|  | 0.099 (-0.43 ↔ 0.63) | 0.71 | -0.58 (-1.1 ↔ -0.48) | **0.033** | -0.079 (-0.84 ↔ 0.68) | 0.84 | -0.015 (-0.062 ↔ 0.032) | 0.53 |
| MRS^f,k^ |  |  |  |  |  |  |  |  |
| Somatic  PP (n=284)  CC (n=289)  ITT (n=368) |  |  |  |  |  |  |  |  |
|  | -0.34 (-0.99 ↔ 0.30) | 0.29 | -0.29 (-0.86 ↔ 0.31) | 0.34 | 0.19 (-0.70 ↔ 1.1) | 0.67 | -0.0081 (-0.062 ↔ 0.046) | 0.77 |
|  | -0.21 (-0.84 ↔ 0.42) | 0.51 | -0.26 (-0.86 ↔ 0.33) | 0.39 | 0.14 (-0.74 ↔ 1.0) | 0.76 | -0.014 (-0.068 ↔ 0.041) | 0.62 |
|  | -0.21 (-0.74 ↔ 0.33) | 0.44 | -0.36 (-0.90 ↔ 0.17) | 0.18 | 0.30 (-0.46 ↔ 1.1) | 0.44 | -0.0074 (-0.055 ↔ 0.040) | 0.76 |
| Urogenital  PP (n=284)  CC (n=289)  ITT (n=368) |  |  |  |  |  |  |  |  |
|  | -0.49 (-1.1 ↔ 0.14) | 0.13 | -0.84 (-1.4 ↔ 0.25) | **0.0056** | 0.55 (-0.33 ↔ 1.4) | 0.22 | 0.025 (-0.029 ↔ 0.078) | 0.37 |
|  | -0.31 (-0.93 ↔ 0.31) | 0.33 | -0.77 (-1.4 ↔ -0.18) | **0.010** | 0.38 (-0.48 ↔ 1.2) | 0.39 | 0.020 (-0.033 ↔ 0.074) | 0.45 |
|  | -0.20 (-0.73 ↔ 0.34) | 0.46 | -0.87 (-1.4 ↔ -0.32) | **0.0017** | 0.37 (-0.40 ↔ 1.1) | 0.35 | 0.032 (-0.015 ↔ 0.080) | 0.19 |
| Psychological  PP (n=284)  CC (n=289)  ITT (n=368) |  |  |  |  |  |  |  |  |
|  | -0.39 (-1.0 ↔ 0.26) | 0.24 | -1.1 (-1.7 ↔ 0.43) | **0.000998** | 0.41 (-0.54 ↔ 1.4) | 0.40 | 0.0047 (-0.057 ↔ 0.058) | 0 99 |
|  | -0.35 (-0.99 ↔ 0.28) | 0.28 | -1.1 (-1.7 ↔ -0.44) | **<0.00099** | 0.43 (-0.50 ↔ 1.4) | 0.36 | -0.0067 (-0.064 ↔ 0.051) | 0.85 |
|  | -0.35 (-0.88 ↔ 0.19) | 0.21 | -1.1 (-1.7 ↔ -0.58) | **<0.0000** | 0.63 (-0.16 ↔ 1.4) | 0.12 | -0.0046 (-0.053 ↔ 0.044) | 0.85 |
| Total  PP (n=284)  CC (n=289)  ITT(n=368) |  |  |  |  |  |  |  |  |
|  | -0.63 (-1.3 ↔ 0.043) | 0.067 | -1.1 (-1.7 ↔ -0.44) | **0.00099** | 0.83 (-0.13 ↔ 1.8) | 0.090 | 0.017 (-0.057 ↔ 0.060) | 0.95 |
|  | -0.43 (-1.1 ↔ 0.22) | 0.19 | -1.1 (1.7 ↔ -0.43) | **0.0012** | 0.73 (-0.22 ↔ 1.7) | 0.13 | -0.0067 (-0.065 ↔ 0.052) | 0.82 |
|  | -0.43 (-0.97 ↔ 0.11) | 0.12 | -1.2 (-1.7 ↔ -0.60) | **<0.0000** | 0.85 (0.61 ↔ 1.63) | **0.035** | -0.0017 (-0.050 ↔ 0.047) | 0.94 |
| ^a^ Dependent variables transformed where improvement is coded as +1 worsening as -1 and unchanged as 0.  ^b^ Short form health survey (SF-36). Physical component score (PCS) and mental component score (MCS). Positive values indicate increase reflect a better Health Related Quality of Life.  ^c^ Per Protocol. Received allocated intervention and responded to follow-up survey.  ^d^ Complete Case. Responded to follow-up survey.  ^e^ Intention To Treat. All participants included with last outcome carried forward (LOCF).  ^f^ Dependent variables transformed where improvement is coded as -1 worsening as +1 and unchanged as 0.  ^g^ Hospital Anxiety and Depression Scale (HADS). Negative values indicate decreased anxiety and depression.  ^h^ Self-rated Exhaustion Disorder (s-ED) identifies risk to develop exhaustion disorder with reduced workability and increased risk of sick leave.  Negative values indicate decreased risk.  ^i^ Perceived Stress Scale 14 (PSS-14). Negative values indicate decreased mental stress.  ^j^ Montgomery-Asberg Depression Rating Scale (MADRS-S) scoring. Negative values indicate decrease in depression.  ^k^ Menopause Rating Scale (MRS) measure prevalence and severity of aging signs and Health-Related Quality of Life. MRS subscale: Somatic symptoms - hot flushes, heart discomfort, sleeping problems and muscle and joint problems,  Psychological symptoms - depressive mood, irritability, anxiety and physical and mental exhaustion, Urogenital symptoms - sexual problems, bladder problems and vaginal dryness, Total score - all subscales added. Negative values indicate improved Health Related Quality of Life. | | | | | | | | |
